# Amino acids protect against methotrexate-induced hepatorenal toxicity in vitro and in vivo

**DOI:** 10.1007/s00210-026-05010-8

**Published:** 2026-02-16

**Authors:** Basma M. Abdelaziz, Hisham A. Nematalla, Ihab T. Abdel-Raheem, Asser I. Ghoneim

**Affiliations:** 1 Department of Pharmacology and Toxicology, Faculty of Pharmacy, Damanhour University, Damanhour, 22514 Egypt; 2 Faculty of Pharmacy and the Research& Innovation Hub, Alamein International University, Alamein, 51718 Egypt; 3 Department of Clinical Pharmacy & Pharmacy Practice, Faculty of Pharmacy and Drug Manufacturing, Pharos University in Alexandria, Canal El Mahmoudia Street, beside Green Plaza Complex, Alexandria, 21648 Egypt

**Keywords:** Methotrexate, Hepatorenal toxicity, Glutamine, Glycine, Methionine, Leucine

## Abstract

Hepatorenal toxicity is one of the most life-threatening adverse effects of methotrexate (MTX). The present study investigated the potential protective effects of glutamine (GLU), glycine (GLY), methionine (MET), and leucine (LEU) in MTX-induced hepatorenal toxicity in vitro and in vivo. Freshly isolated hepatocytes and renal slices of rats were used to investigate the effect of these amino acids in vitro, while male Sprague–Dawley rats were used to evaluate their effects in vivo. Rats were assigned into 6 equal groups: a negative control, MTX (20 mg/kg/day, i.p., 3 days), MTX + (GLU 25 mg/kg/day i.p., 6 days), MTX + (GLY 0.5 gm/kg/day, i.p., 3 days), MTX + (MET 1 mg/kg/day, i.p., 6 days) and MTX + LEU (50 mg/kg/day, i.p., 6 days). These amino acids showed enhanced viability of liver and kidney cells assessed by trypan blue exclusion and lactate dehydrogenase leakage tests, respectively. Besides, pre-incubation of suspended hepatocytes or renal slices with GLU (25 mM and 10 mM, respectively), GLY (25 mM and 5 mM, respectively), MET (12.5 mM and 5 mM, respectively) or LEU (25 mM and 10 mM, respectively), 30 min before MTX, significantly down-regulated caspase-3 and TNF-α levels as compared with MTX-intoxicated groups. MTX-induced hepatorenal toxicity was manifested by increasing liver and kidney enzymes, oxidative stress and severe histopathological alterations. Amino acids pre-treatment abrogated MTX-induced alteration in hepatorenal toxicity indices as evidenced by amelioration in oxidative stress, inflammatory mediators, and histopathological changes. Amino acids may serve as a promising adjuvant therapy with MTX as it may ameliorate its hepatorenal toxicity through antioxidant, anti-inflammatory and anti-apoptotic mechanisms.

**Supplementary Information:**

The online version contains supplementary material available at 10.1007/s00210-026-05010-8.

## Introduction

MTX is a folic acid antagonist and a cytotoxic antineoplastic drug widely used in the treatment of various malignancies and autoimmune diseases (Koźmiński et al. 2020). Unfortunately, its effectiveness is limited by the lack of selectivity, causing serious adverse effects, especially on liver and kidneys (Howard et al. 2016). MTX causes hepatotoxicity through different mechanisms including excessive generation of reactive oxygen and nitrogen species (ROS/RNS), decreasing the cellular anti-oxidant defense capacity, production of pro-inflammatory cytokines, and enhancing lipid peroxidation that damages hepatocytes membrane (Conway and Carey 2017; Ezhilarasan 2021).

On the other hand, renal toxicity is a life-threatening adverse effect associated with long term and/or high dose of MTX treatment. More than 90% of MTX is excreted through the kidneys (Wang et al. 2018). The deposition of MTX and its metabolite, 7-OH MTX, in urine contributes to intratubular obstruction, impaired renal function and subsequent toxicity (Wang et al. 2018). Hepatorenal toxicity of MTX-induced is mediated by inflammation, oxidative stress, and apoptosis (El-Sheikh et al. 2015). Therefore, agents which can modulate inflammation, oxidative stress, and apoptosis without diminishing MTX’s efficacy may offer a promising strategy to mitigate its toxicity. One such strategy is the addition of different amino acids that may enhance safety and patient satisfaction.

Many studies have shown the benefits of amino acids in several clinical conditions. Amino acids are not only building blocks for synthesis of protein; moreover, they act as modulators of cell functions as well as drugs (Ghoneim 2012; Hassan et al. 2021). GLU is the most abundant free amino acid in the human body which acts as a precursor for nucleotide synthesis in different cell types (Hu et al. 2022). It serves as a main fuel for kidneys and liver under physiological conditions (Kim et al. 2015). GLY is the smallest, most important and simple non-essential amino acid. It is synthesized from choline, hydroxyproline, serine, and threonine via inter-organ metabolism in which liver and kidneys are mainly involved (Razak et al. 2017). GLU and GLY possess anti-inflammatory, anti-apoptotic, and immunomodulatory properties (Ghoneim 2012). MET is considered one of the most easily oxidizable amino acids, involved in protein synthesis. MET possesses antioxidant activities and effectively scavenge free radicals (Azad et al. 2018). LEU is a branched chain amino acid (BCAA) that plays a key role in protein biosynthesis. The potential therapeutic activities of BCAAs in liver and kidney diseases have gained great attention (Macotela et al. 2011; Vahdatpour et al. 2020).

Generally, in vitro investigations help in predicting efficacy and potency in a controlled, isolated environment using multiple concentrations in a short experimental time, regarding 3Rs principle. It is firstly conducted to determine non-toxic effective concentrations and to elucidate potential cellular mechanisms including inflammation and apoptosis, while in vivo experiments confirm the systemic relevance of the observed cellular effects, because they reflect the physiological exposure with different body cell types (Boraschi and Italiani 2016). Therefore, combining in vitro and in vivo approaches bridge the gap and provide a comprehensive understanding from cellular to systemic level.

The aim of the present study is to investigate the potential protective effects of the non-essential amino acids (glutamine and glycine), the essential amino acid methionine, and BCAA leucine in MTX-induced hepatorenal toxicity. Firstly, in vitro experiments assessed the hepatoprotective and renoprotective mechanisms of the amino acids using freshly isolated hepatocytes and renal slices from rats. Secondly, in vivo studies evaluated oxidative stress and histopathological alterations to validate the in vitro findings under physiological conditions.

## Materials and methods

### Chemicals

Collagenase type I and trypan blue were purchased from Alpha Aesar Germany. Methotrexate was from Shanxi PUDE Pharmaceutical Co., Ltd., Datong, Shanxi China. HEPES [N-(2-hydroxyethyl) piperazine-N′−2-ethanesulfonic acid] was purchased from Fisher BioReagents USA. Thiobarbituric acid (TBA), sulfanilamide (SNA), reduced glutathione (GSH), tetramethoxypropane (TMP) standard solution, 5,5-dithiobis (2-nitrobenzoic acid) DTNB, vanadium III chloride (VCL_3_), thiopental, and fetal bovine serum (FBS) were purchased from Sigma-Aldrich (St. Louis, USA). Bovine albumin fraction v was purchased from MP Biomedicals USA. Dulbecco’s modified Eagle medium (DMEM) and Penicillin G 100 U/ml and Streptomycin 100 µg/ml were purchased from Lonza (Basel, Switzerland). Acetonitrile was purchased from Scharlab. Spain. N-(1-Naphthyl) ethylenediamine dihydrochloride (NED) was from Fluka Chemie AG. Switzerland. l-Glutamine and l-methionine were from Research-Lab Fine Chem Industries (Mumbai, India). l-Leucine was purchased from Acros Organics (Belgium, Europe). Glycine purchased from Alpha Chemika (India). All other chemicals were of analytical grade from local suppliers.

### Experimental design for hepatocytes in vitro isolation

#### Hepatocyte isolation and culture

Seven male Sprague–Dawley rats, 250–300 g, obtained from The Nile Co. for Pharmaceuticals and Chemical Industries (Cairo, Egypt), were used to obtain hepatocytes cells. Freshly isolated rat hepatocytes were prepared using collagenase techniques of perfusion and digestion of rat liver using (peristaltic pump; E-Chrom Tech Co. Ltd., Model B07) (Berry and Friend 1969; Fry 1981; Seglen 1976), with minor modifications as described previously (Ghoneim 2009; Khatab et al. 2022). First, rats were anesthetized with an i.p. injection of thiopental sodium (40 mg/kg) (Dogan et al. 2010). The peritoneal cavity was incised, the portal vein was successfully cannulated, and the inferior vena cava was transected. The liver was rapidly perfused with preperfusion Krebs–Ringer (KR) buffer. Then, the thorax was incised, and outflow tubing was gently inserted into the inferior vena cava. A three-way valve was redirected to an identical buffer supplemented with collagenase (type I, 0.05%), 0.5% BSA, and calcium chloride (CaCl2; 2 mM). Recirculation was allowed for an additional 5 to 15 min to ensure adequate perfusion and tissue digestion.

The liver was then placed in an ice-cold modified Krebs–Henseleit (KH) incubation solution. Following the gentle disruption of the digested liver, the dilute solution was filtered through a 100 μm mesh and then centrifuged twice for 1 min at 1600 rpm. Before use, isolated hepatocytes in suspension were incubated on ice for about 60 min. The suspension was thereafter removed from cold storage and incubated for 3 min at 37 °C to adapt to the new temperature before treatment. The final pellet represented the working suspension of isolated, purified hepatocytes, largely free of non-parenchymal cells, and debris that had seeped from injured cells. After that, 2-ml cell suspension in a caped vessel with a surface area of the incubation medium of 4–5 cm^3^ 10-ml amber glass vials were used. Cells at a concentration of 5 × 10^6^ cells/ml were incubated for 120 min in Krebs–Henseleit buffer (KH) supplemented with 10 mM HEPES and 0.2% BS in a shaking water bath (Daihan-Digital Precise Shaking Water Bath MaXtur dyTM18) at 100 oscillations per minute (opm) at 37 °C and pH 7.4. The viability of the cells was determined by counting the percentage of hepatocytes which were able to exclude trypan blue dye.

#### Experimental design

**Fig. 1:**
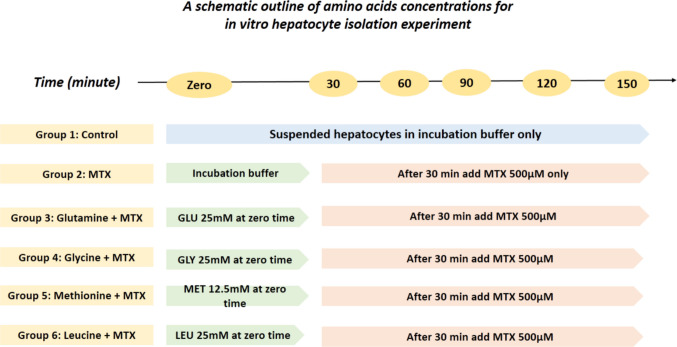
a schematic diagram of amino acids concentrations for hepatocyte isolation experiment. Glutamine (GLU 25 mM), glycine (GLY 25 mM), methionine (MET 12.5 mM), or leucine (LEU 25 mM) were added 30 min prior to methotrexate (MTX 500 μM)

All tested amino acids (GLU, GLY, MET, or LEU) were added 30 min prior to MTX (500 μM). MTX was incubated for 2 h to ensure sufficient exposure to reach LD50 (Khatab et al. 2022). Suspension cultures of hepatocytes (5 × 10^6^ cells/ml) were incubated as shown in Fig. 1 as follows: group I (control cells in the incubation buffer only), group II (cells received MTX 500 μM only), group III (cells received GLU 25 mM + MTX), group IV (cells received GLY 25 mM _+_ MTX), group V (cells received MET 12.5 mM + MTX) and group VI (cells received LEU 25 mM + MTX). (3–7) Distinct hepatocyte preparations were performed for each group (one for each rat), each done in three technical replicates.

#### Cell viability assay (trypan blue exclusion test)

Cells from suspension cultures were examined within 1 min following mixing an equal volume with the solution of trypan blue dye to a final concentration of 0.2% using a standard Neubauer hemocytometer under a light microscope (Ghoneim 2009). Cell morphology was examined immediately after isolation, and the suspensions displaying the typical polygonal hepatocyte morphology with intact nuclei and clear cytoplasm and well defined cell border were only used. In contrast, damaged cells are flattened, irregular in shape, and take up trypan blue (Lefkowitch 2020). Initial cell viability was more than 75% of freshly isolated hepatocytes which normally excluded trypan blue. Cell viability was assessed at many time intervals: 0.5, 1, 1.5, 2, and 2.5 h throughout the incubation period and the percentage staining with the dye was calculated using the following equation (Strober 1997).

$$\text{Viability }({\%})=\frac{[\text{Number of cells of nonstained nuclei}]}{[\text{Number of cells of stained nuclei}+\text{number of cells of nonstained nuclei}] }\times 100$$

#### Measurement of caspase‑3 activity

Caspase-3 activity was measured in the hepatocyte cell lysate by the use of Sigma’s caspase-3 colorimetric assay kit according to the manufacturer protocol. It relies on the formation of the chromophore p-nitroaniline (p-NA) by cleavage from the labeled substrate DEVD-pNA. The p-NA can be quantified using a spectrophotometer at 405 nm (Strober 1997).

#### Measurement of pro‑inflammatory cytokines (TNF‑α) level

This assay is performed on hepatocytes suspension by the use of Quantikine® colorimetric sandwich ELISA kit (Catalog Numbers RTA00, SRTA00, and PRTA00), was utilized according manufacturer protocol. It relies on a quantitative sandwich enzyme immunoassay technique using a rat TNF-α monoclonal antibody.

#### Total protein content

Total protein content in hepatocytes suspension was measured using pierce™ bicinchoninic (BCA) protein assay kit according to the manufacturer protocol. The assay is based on the reduction of Cu^+2^ to Cu^+^ by protein in an alkaline medium and colorimetric detection of the cuprous cation (Cu^+^) using bicinchoninic acid. The absorbance of the resultant purple product was determined at 562 nm using a plate reader (Brown et al. 1989).

### Experimental design for renal slices in vitro preparation

#### Preparation and incubation of renal cortical slices

Seven male Sprague–Dawley rats, 250–300 g, were used to prepare renal cortical slices as previously described (Obatomi et al. 1998; Zhang et al. 1992). After fasting overnight, rats were anaesthetized by thiopental sodium (40 mg/kg, i.p) (Dogan et al. 2010), to minimize pain and then euthanized by increasing the anesthetic dose three times according to AVMA guidelines, the kidneys were rapidly removed, decapsulated and placed into ice-cold saline. The slices about (0.3–0.5 mm thickness) were obtained using a razor blade on a Petri plate in an ice-bath. Following preparation of all slices, they were blotted dry to remove excess medium. Renal slices weighing (150–200) mg were transferred to 25-ml stoppered Erlenmeyer flask with cap at 37 °C on a horizontal shaker at (100 opm) and allow to stabilize for 10–15 min. Each flask contained 5-ml incubation medium (134 mM NaC1, 5.9 mM KC1, 1.5 mM CaCI_2_, 1.2 mM MgCI_2_, 11.5 mM glucose, and 5.8 mM HEPES buffer, pH 7.4, gassed with (95% O_2_:5% CO_2_)). Slices were incubated in 3.2-ml Williams medium E, pre-warmed at 37 °C, with penicillin (100 U/ml) plus streptomycin (100 µg/ml) and supplemented with glucose (final concentration 25 mM) (Kanter et al. 2002).

#### Experimental design

**Fig. 2:**
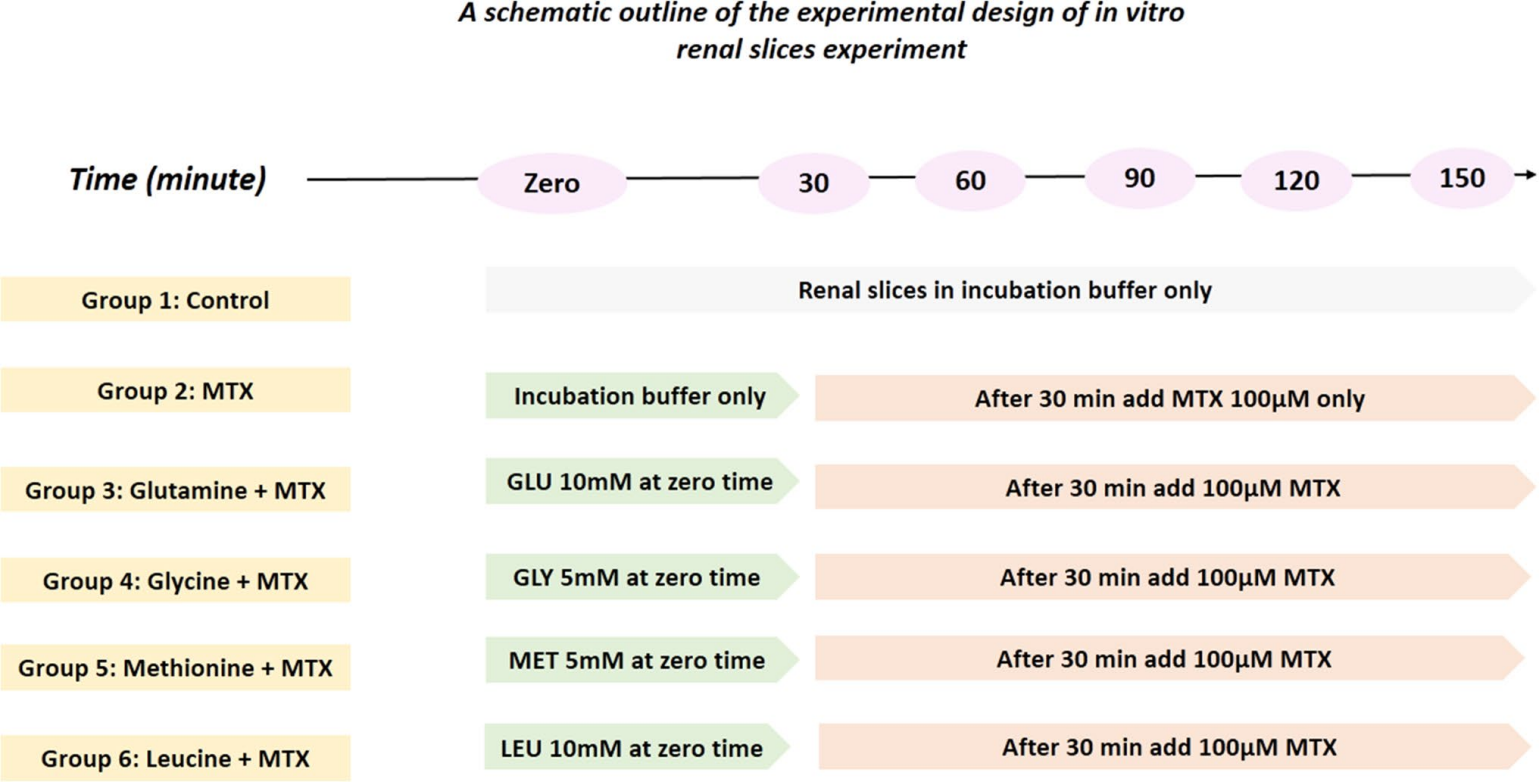
A schematic diagram of amino acids concentrations for renal slices experiment. Glutamine (GLU 10 mM), glycine (GLY 5 mM), methionine (MET 5 mM), or leucine (LEU 10 mM) were added 30 min prior to methotrexate (MTX 100 μM)

All tested amino acids (GLU, GLY, MET or LEU) were added 30 min prior to MTX (100 μM) (Caetano-Pinto et al. 2017). Renal slices were incubated as illustrated in Fig. 2 as follows: group I (control cells in the incubation buffer only), group II (cells received MTX 100 μM only), group III (cells received GLU 10 mM + MTX), group IV (cells received GLY 5 mM + MTX), group V (cells received MET 5 mM + MTX) and group VI (cells received LEU 10 mM + MTX). (3–7) Distinct renal slice preparations were performed for each group (one for each rat), each done in three technical replicates. Several concentrations of amino acids were used in pilot studies (supplementary data) in hepatocytes suspension cultures and renal slices to select the least concentration of each amino acid that gave a significant protective effect and beyond which no further beneficial effects is noticed, to be used in the following biochemical tests (Bretz et al. 2010). From a pharmacological point of view, selecting the minimum effective concentration is preferable because it reduces the risk of toxicity, diminishes unnecessary drug exposure, and enhances overall safety (Dimmitt et al. 2017). Different concentration ranges of amino acids were used for hepatocyte suspensions and renal slice preparations due to the differences in tissue architecture, diffusion properties, uptake, metabolic response and sensitivity to MTX and amino acids. Hepatocytes have high enzymatic activity and greater permeability, while renal slices are more diffusion-limited and metabolically distinct (Dumas et al. 2021; Sanz-García et al. 2021).

#### Lactate dehydrogenase leakage

LDH leakage was used as common method to assess cells integrity in renal slices (Zhang et al. 1992). LDH release was assessed in the incubation medium by a colorimetric enzymatic assay according to the manufacturer protocol using cytotoxicity detection kits from Spectrum from Approaches of Bioscience. LDH leakage was expressed as units per liter (U/l).

#### Measurement of caspase‑3 activity

Caspase-3 activity was measured in the renal slice homogenates by the use of Sigma’s caspase-3 colorimetric assay kit according to the manufacturer protocol. It relies on the formation of the chromophore p-nitroaniline (p-NA) by cleavage from the labeled substrate DEVD-pNA. The p-NA can be quantified using a spectrophotometer at 405 nm (Nicholson et al. 1995).

#### Measurement of pro‑inflammatory cytokines (TNF‑α) level

This assay is performed on renal slices homogenate by the use of QuantikineR colorimetric sandwich ELISA kit according manufacturer protocol. It relies on a quantitative sandwich enzyme immunoassay technique using a rat TNF-α monoclonal antibody.

#### Total protein content

Total protein content in renal slices was measured using pierce™ bicinchoninic (BCA) protein assay kit according to the manufacturer protocol. The assay is based on the reduction of Cu^+2^ to Cu^+^ by protein in an alkaline medium and colorimetric detection of the cuprous cation (Cu^+^) using bicinchoninic acid. The absorbance of the resultant purple product was determined at 562 nm using a plate reader (Brown et al. 1989).

### Experimental design for in vivo studies

#### Animals

Experiments were carried out on adult male Sprague–Dawley rats weighing 250**–**300 g, obtained from The Nile Co. for Pharmaceuticals and Chemical Industries (Cairo, Egypt). Animals were kept in our animal facility (Faculty of Pharmacy, Damanhour University, Damanhour, Egypt). Animals were housed in standard ventilated stainless-steel cages at ambient temperature of 25 ± 5 °C with a 50–60% relative humidity. They were fed a standard diet of pellets and tap water ad libitum. Rats were allowed to acclimatize to laboratory conditions for at least 1 week prior to any experiments. Animals received humane care in compliance with institutional guidelines and were sacrificed between 9 A.M. and 11 A.M. All procedures performed in this research have followed accepted principles of ethical and professional conduct according to approval reference number (1122PO31) by the Research Ethics Committee of the Faculty of Pharmacy, Damanhour University, regarding originality, risk control, and community services.

#### Experimental design

**Fig. 3:**
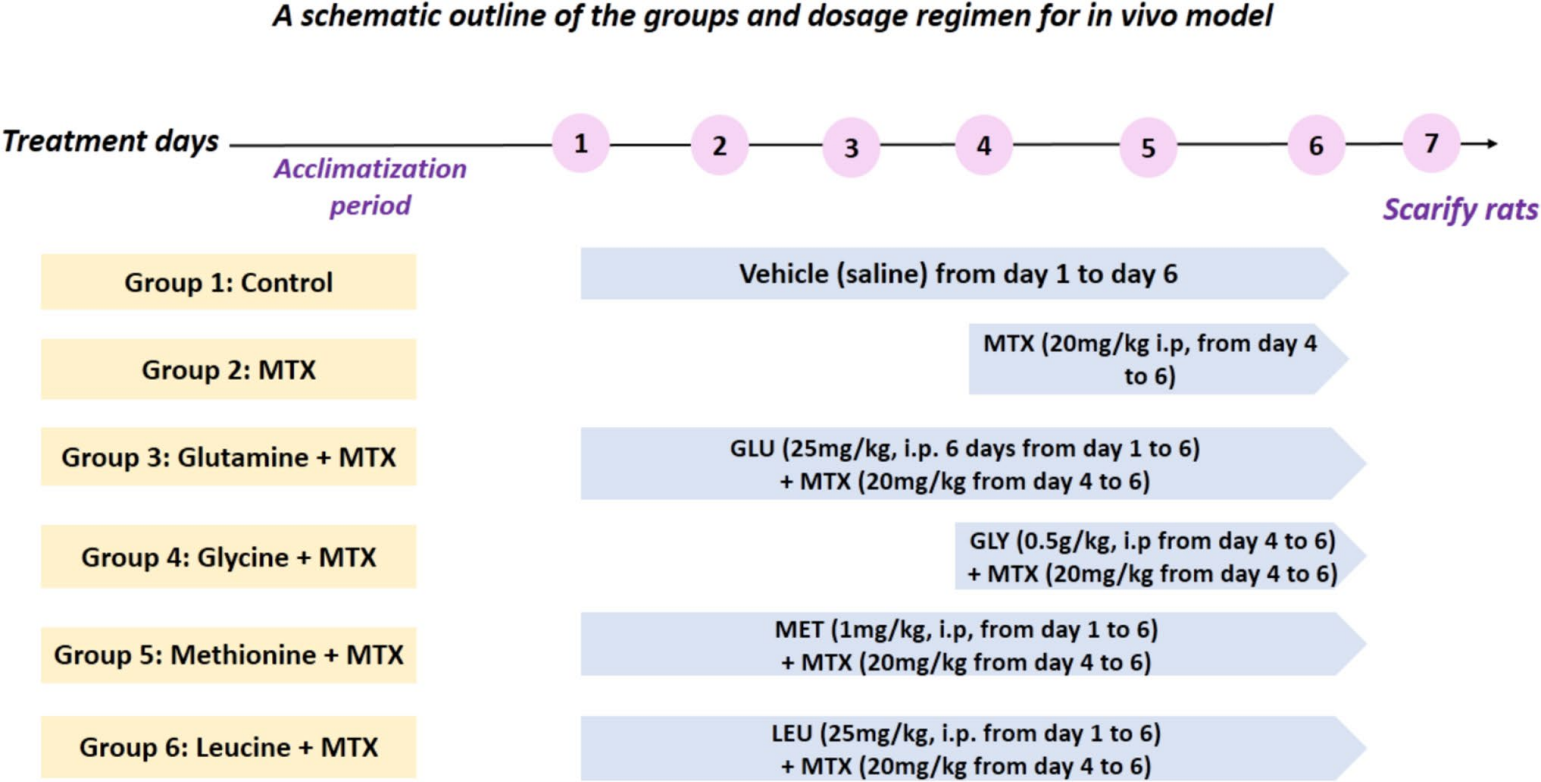
A schematic diagram of amino acids concentrations for the in vivo model. Glutamine (GLU 25mg/kg/day, i.p., 6 days), glycine (GLY 0.5 gm/kg/day, i.p., 3 days), methionine (MET 1 mg/kg/day, i.p., 6 days), or leucine (LEU 50 mg/kg/day, i.p., 6 days) were injected 60 min prior to methotrexate (MTX 20 mg/kg/day, i.p., 3 days)

Thirty-six healthy rats were randomly divided into six groups, each of six. Experimental grouping was done as demonstrated in Fig. 3. Rats in group I served as a negative control, received saline only during the whole experimental period. Rats in group II received MTX only at (20 mg/kg/day, i.p.) for 3 consecutive days (Al-Azem et al. 2019; Bu et al. 2018). MTX was administrated from the fourth to the sixth day of the start of the experiment (MTX LD_50_ = 60 mg/kg (Ermens et al. 1989)). Rats in groups III received GLU at (25 mg/kg/day, i.p.) for 6 consecutive days from day 1 to day 6 (Schemitt et al. 2019). Rats in group IV received GLY (0.5 gm/kg/day, i.p) for 3 consecutive days from day 4 to day 6, dose of glycine was chosen according to previous studies (Jacob et al. 2003; Senthilkumar et al. 2004). Group V rats received MET (1 mg/kg/day, i.p.) for 6 consecutive days from day 1 to day 6 (Ayhanci et al. 2010). The sixth group VI received LEU (50 mg/kg/day. i.p.) for 6 consecutive days from day 1 to day 6 (Walker et al. 2019). Groups from III to VI received MTX (20 mg/kg, i.p.) from day 4 to day 6. All amino acids doses were given 60 min prior to the administration of MTX. Twenty-four hours after the last dose of MTX, animals were weighed, anesthetized by thiopental sodium (40 mg/kg, i.p) (Dogan et al. 2010); the blood was collected via cardiac puncture, immediately after blood collection; rats were euthanized by increasing the anesthetic dose three times according to AVMA guidelines. Then, livers and kidneys were removed. The liver and the kidneys were dissected into two parts: the 1 st part was fixed with 10% formalin for histopathological examination and the 2nd part was used for biochemical analysis.

#### Biomarkers of hepatic and renal toxicity assessment

Serum assessment of aspartate transaminase (AST), alanine transaminase (ALT), creatinine, urea, and blood urea nitrogen (BUN) levels was performed spectrophotometrically. These biomarkers were assayed according to manufacturer protocol using available commercial kits from Bi-Step ARKAN Science Technology.

#### Preparation of tissue homogenates

The liver and kidneys were homogenized in ice cold phosphate buffer (0.01 M, pH 7.4), to prepare 10% w/v homogenate. The homogenate was centrifuged at 3000 rpm for 20 min and the supernatant was separated to avoid sample thawing and was kept at – 80 °C until use (Mahmoud et al. 2018; Noeman et al. 2011).

#### Determination of lipid peroxidation levels (MDA) in liver and kidney tissues

The aliquot of the liver and kidney homogenates were used to assess MDA levels as previously described (Ohkawa et al. 1979). This method is based on the formation of a pink compound with thiobarbituric acid-reactive substances (TBARS). The absorbance of the colored complex was measured at 532 nm. MDA concentration was measured using a standard curve equation and results were expressed as nmol/g tissue.

#### Estimation of reduced glutathione levels (GSH) in liver and kidney tissues

GSH was determined by the spectrophotometric method using the Ellman’s reagent (Ellman 1959). The method is based on the reduction of DTNB to produce a yellow compound and its absorbance was measured at 412 nm. GSH concentration was measured using a standard curve equation and results were expressed as μg/g tissue.

#### Determination of nitric oxide level in liver and kidneys tissues

Total nitric oxide (NO) levels were measured after the conversion of nitrate to nitrite by nitrate reductase (Griess diazotization reaction) as described (Miranda et al. 2001). The absorbance of the pink-colored product was measured spectrophotometrically at 540 nm. The concentration of nitrite was determined using a sodium nitrite standard curve and the findings were expressed as (nmol/mg tissue).

#### Histopathological examination

Following necropsy, liver and kidney tissue from different groups were collected and immediately fixed in phosphate-buffered formalin (10%, pH 7.4) for at least 24 h, which were then processed using conventional paraffin embedding technique. Sections of 5 μm thick were sliced, mounted on slides deparaffinated in xylene and rehydrated using decreasing concentrations of ethanol. Slides were stained with hematoxylin and eosin (H&E) for routine histopathological setting. Stained sections were blindly observed and evaluated using light microscope (Leica, DM500) and photographed at magnification powers of 100 × and 400 × using a digital camera (EC3, Leica, Germany) (Bancroft and Gamble 2008). A histological lesion scoring approach was adopted to show the severity of histopathological lesions. In each animal group, 5 H&E-stained slides (one slide/rat) were examined, and 10 random fields per slide were used for grading the various pathological lesions in hepatic and renal tissues in a blinded vision. The severity of pathological lesions was evaluated using the following semi-quantitative scoring system as: (−) absence of the lesion = normal histology with zero involvement of the examined field, (+) mild = 5–25% of the tested field was involved, (+ +) moderate = 26–50% of the examined field was involved, and (+ + +) severe damage ≥ 50% of the examined tissue sections were involved depending on the number of affected slides and regions within the same slide (Alam et al. 2021).

### Statistical analysis

Data are expressed as the mean ± standard error of the mean (SEM). A one-way analysis of variance (ANOVA) followed by post hoc Tukey’s test was carried out to analyze multiple comparisons. Tests were done by Graph Pad Prism® software package version 9 (GraphPad Software Inc., CA, USA), and the differences were considered at *p* < 0.05, *p* < 0.01, *p* < 0.001, and *p* < 0.0001, corresponding to significant, highly significant, very highly significant and extremely significant, respectively.

## Results

### Results of in vitro hepatocytes suspension cells

#### Effects of different amino acids on MTX-induced cytotoxicity in hepatocytes suspension

**Fig. 4:**
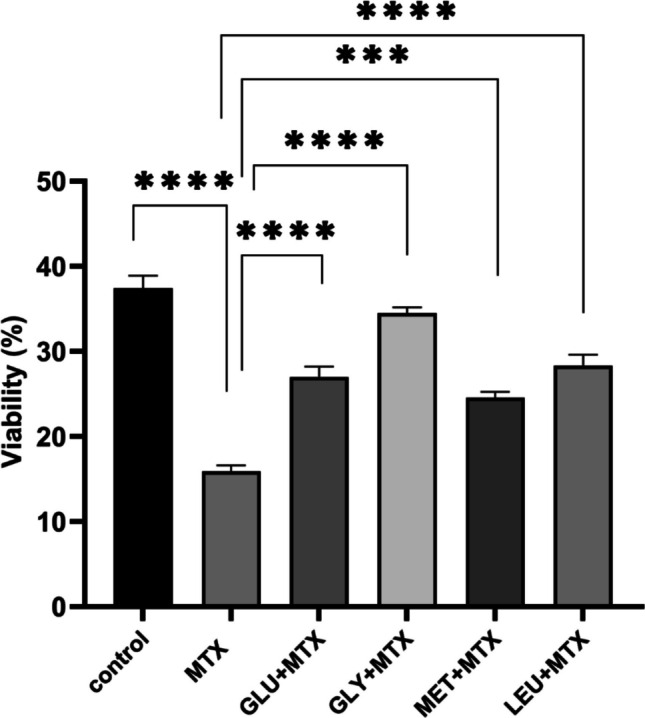
Effects of glutamine (GLU), glycine (GLY), methionine (MET), or leucine (LEU) on methotrexate (MTX)–induced trypan blue (TB) uptake in suspended hepatocytes. Values are represented as means ± SEM for (3–7) hepatocytes preparations. Samples were taken for TB estimation after 150-min incubation (37 °C, 100 rpm). Statistically significant difference among groups is indicated as ****p < 0.0001, ***p < 0.001. Statistical analysis was done using analysis of variance (ANOVA) followed by Tukey as post hoc test for multiple comparisons

The trypan blue exclusion test was used to assess the ability of hepatocytes to keep their viability with MTX alone or in combination with GLU, GLY, MET or LEU. Figure 4 illustrates that incubation of hepatocytes with MTX (500 μM) for 120 min induced a 57.59% decrease in cell viability compared to control group (*p* < 0.0001). However, pre-incubation of hepatocytes with GLU (25 mM), GLY (25 mM), MET (12.5 mM), or LEU (25 mM) 30 min before addition of MTX reduced the cell death by 13.25% (*p* < 0.0001), 22.04% (*p* < 0.0001), 10.31% (*p* < 0.001), and 14.84% (*p* < 0.0001), respectively, as compared to the MTX group.

#### Effects of different amino acids on MTX-induced Casp-3 activation in hepatocytes suspension

**Fig. 5:**
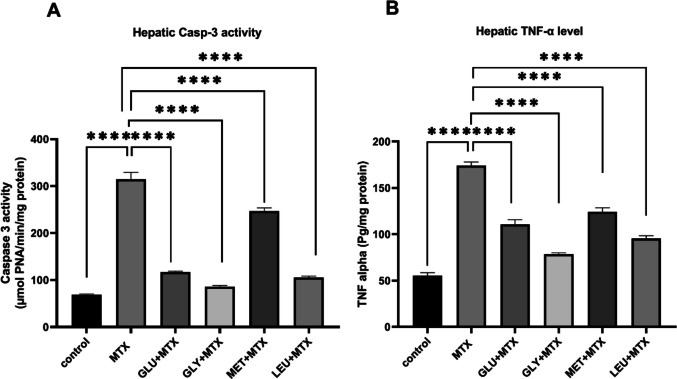
Effects of glutamine (GLU), glycine (GLY), methionine (MET), or leucine (LEU) on methotrexate (MTX)–induced elevation in caspase-3 activation (**A**) and tumor necrosis factor alpha (TNF-α) level (**B**) in suspended hepatocytes. Values are represented as means ± SEM for (3–7) separate preparations. Statistically significant difference among groups is indicated as ****p < 0.0001. Statistical analysis was done using analysis of variance (ANOVA) followed by Tukey as post hoc test for multiple comparisons

Caspase-3 is a sensitive and specific biomarker that play a key role in apoptosis. Data in Fig. 5A illustrates that incubation of hepatocytes with MTX (500 μM) caused significant increase (*p* < 0.0001) in Casp-3 activation by more than three folds as compared with control values. However, pre-incubation of hepatocytes with GLU (25 mM), GLY (25 mM), MET (12.5 mM), or LEU (25 mM) 30 min prior to addition of MTX reduced casp-3 activation (*p* < 0.0001) by 62.83%, 72.84%, 21.53%, and 66.48%, respectively, as compared with MTX-treated hepatocytes.

#### Effects of different amino acids on MTX-induced elevation of tumor necrosis factor-alpha (TNF-α) in hepatocytes suspension

TNF-α is a pro-inflammatory cytokine. Data in Fig. 5B shows that incubation of hepatocytes with MTX (500 μM) caused a significant elevation (*p* < 0.0001) in the TNF-α level by more than two folds as compared with control values. However, pre-incubation of hepatocytes with GLU (25 mM), GLY (25 mM), MET (12.5 mM), or LEU (25 mM), 30 min prior to addition of MTX, reduced TNF-α level (*p* < 0.0001) by 36.44%, 54.95%, 28.7%, and 45.19%, respectively, as compared with MTX-treated hepatocytes.

### Results of in vitro renal slices technique

#### Different amino acids ameliorated lactate dehydrogenase leakage induced by MTX in renal slices

**Fig. 6:**
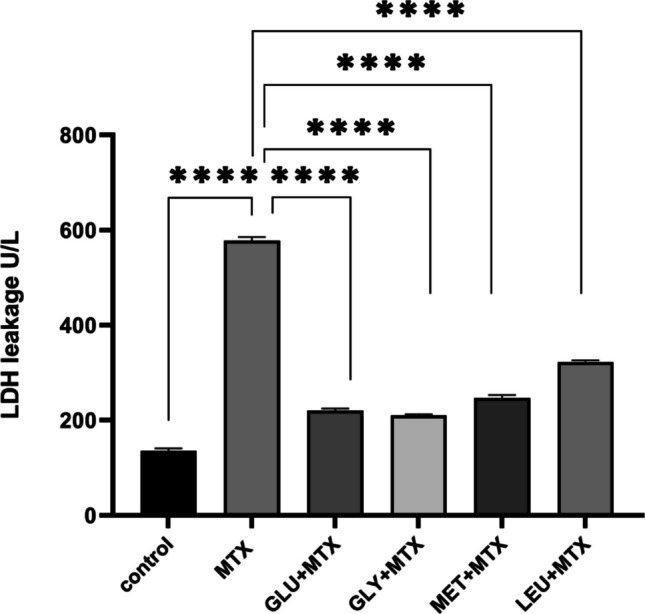
Effects of glutamine (GLU), glycine (GLY), methionine (MET), or leucine (LEU) on methotrexate (MTX)–induced lactate dehydrogenase (LDH) leakage in renal slices of rats. Values are represented as means ± SEM for (3–7) renal slices preparations. Samples were taken for LDH release determination after 150 min incubation (37 °C, 100 rpm). Statistically significant difference among groups is indicated as ****p < 0.0001. Statistical analysis was done using analysis of variance (ANOVA) followed by Tukey as post hoc test for multiple comparisons

LDH leakage is a biomarker for loss of membrane integrity, in a time dependent manner. Data in Fig. 6 illustrates that incubation of renal slices with MTX (100 μM) for 120 min significantly increased (*p* < 0.0001) LDH leakage of renal slices by more than three folds as compared with control values. However, pre-incubation of renal slices with GLU (10 mM), GLY (5 mM), MET (5 mM), or LEU (10 mM), 30 min prior to addition of MTX decreased LDH leakage (*p* < 0.0001) by 61.82%, 63.56%, 57.19%, and 44.27%, respectively, as compared with MTX-treated slices.

#### Effects of different amino acids on MTX-induced apoptosis in renal slices cells

**Fig. 7:**
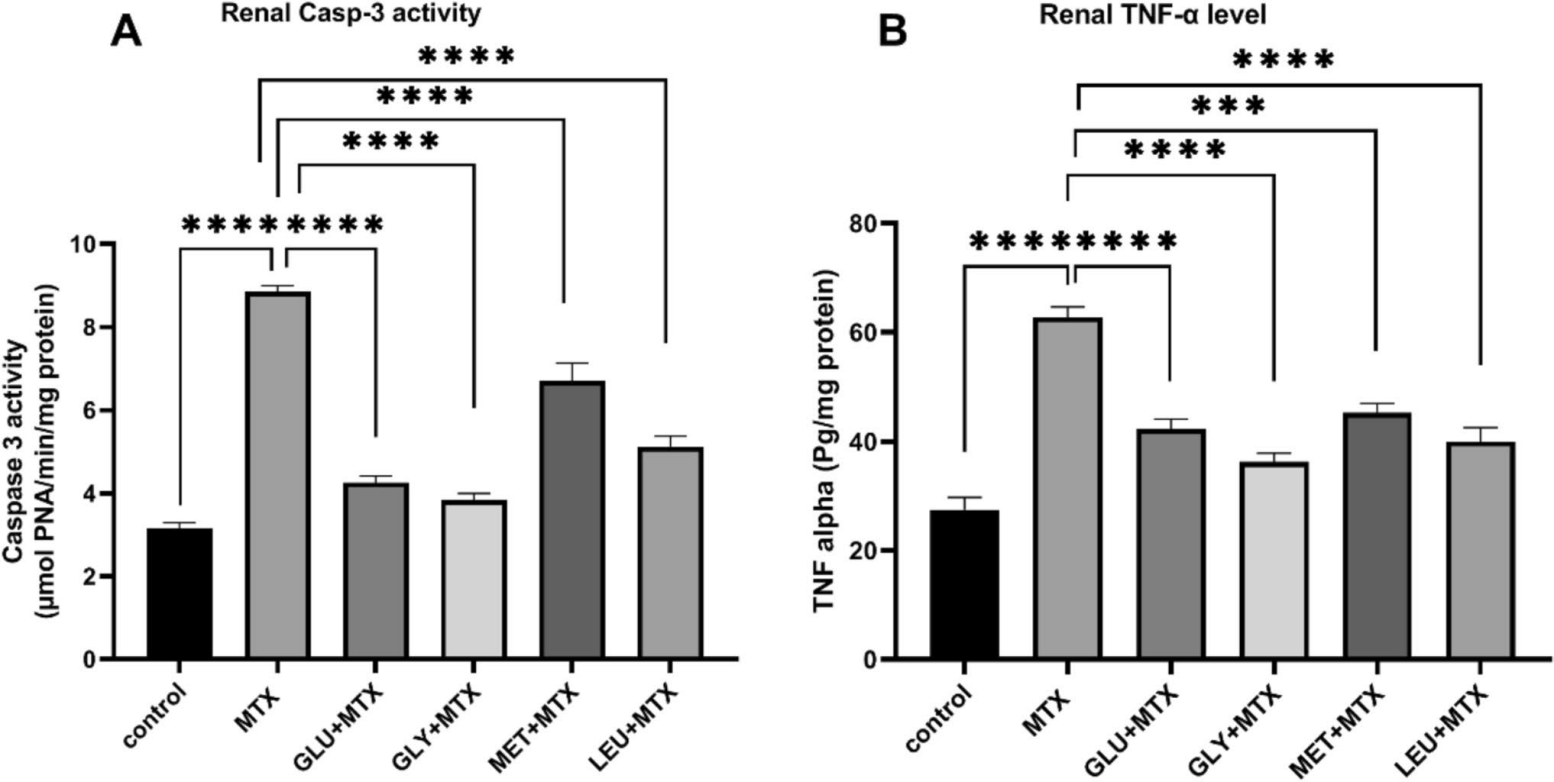
Effects of glutamine (GLU), glycine (GLY), methionine (MET), or leucine (LEU), on methotrexate (MTX)–induced elevation in caspase-3 activation (**A**) and tumor necrosis factor alpha (TNF-α) level (**B**) in renal slices. Values are represented as means ± SEM for (3–7) separate preparations. Statistically significant difference among groups is indicated as ****p < 0.0001. Statistical analysis was done using analysis of variance (ANOVA) followed by Tukey as post hoc test for multiple comparisons

As illustrated in Fig. 7A incubation of renal slices with MTX (100 μM) caused a significant increase (*p* < 0.0001) in Casp-3 activation by about two folds as compared with control slices. However, pre-incubation of renal slices with GLU (10 mM), GLY (5 mM), MET (5 mM), or LEU (10 mM), before addition of MTX reduced casp-3 activation (*p* < 0.0001) by 51.98%, 56.72%, 24.29%, and 42.03%, respectively, as compared with MTX-treated slices.

#### Effects of different amino acids on MTX-induced elevation of tumor necrosis factor-alpha (TNF-α) in kidney slices cells

Data in Fig. 7B shows that incubation of renal slices with (100 μM) of MTX caused significant elevation (*p* < 0.0001) in the TNF-α level by more than one fold as compared with control values. However, pre-incubation of renal slices with GLU (10 mM), GLY (5 mM), MET (5 mM), or LEU (10 mM), before MTX addition, reduced TNF-α level by 32.67% (*p* < 0.0001), 42.23% (*p* < 0.0001), 27.89% (*p* < 0.001), and 36.26% (*p* < 0.0001), respectively, as compared with MTX-treated renal slices.

### Results of in vivo biochemical tests

#### In vivo hepatic and renal protective effects of different amino acids on hepatorenal toxicity induced by MTX

**Fig. 8:**
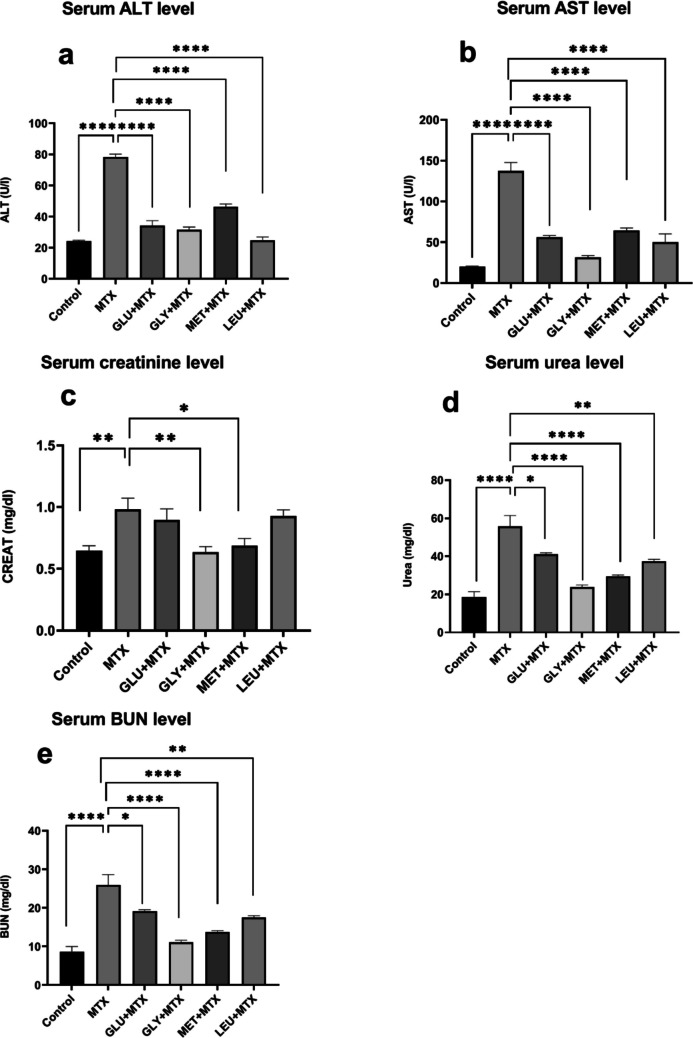
Effects of glutamine (GLU), glycine (GLY), methionine (MET), or leucine (LEU) on the levels of SGPT (**a**), SGOT (**b**), creatinine (**c**), urea (**d**), and BUN (**e**) in the serum of rats intoxicated with methotrexate (MTX). Values are represented as means ± SEM. *n* = 6 per group. Statistically significant difference among groups is indicated as ****p < 0.0001, **p < 0.01, and *p < 0.05. Statistical analysis was done using analysis of variance (ANOVA) followed by Tukey as post hoc test for multiple comparisons

To examine the effect of the four amino acids (GLU, GLY, MET, and LEU) on the hepatic and renal injury following administration of MTX in rats, the levels of serum ALT and AST were assessed to ensure hepatic injury, while serum levels of creatinine, urea, and BUN were measured to ascertain renal damage. Figure 8 illustrates the effect of the four amino acids on the serum biomarkers of hepatic and renal toxicities in MTX-treated rats. Intraperitoneal administration of MTX (20 mg/kg, for 3 consecutive days), significantly increased (*p* < 0.0001) the activities of ALT (Fig. 8a) and AST (Fig. 8b) by more than onefold as compared with control rats. However, pretreatment with GLU (25 mg/kg for 6 consecutive days) decreased the elevation of ALT and AST induced by MTX (*p* < 0.0001) by 64.1% and 59.77%, respectively. Also, co-administration of GLY (0.5 g/kg for 3 consecutive days) decreased MTX-induced elevation in of ALT and AST levels (*p* < 0.0001) by 93.2% and 93.50%, respectively. Besides, pretreatment of rats with MET (1 mg/kg for 6 consecutive days), decreased the MTX-induced elevation in ALT and AST levels (*p* < 0.001) by 50.47% and 47.39%, respectively. Moreover, pretreatment of rats with LEU (50 mg/kg for 6 consecutive days), decreased the elevation of ALT and AST induced by MTX (*p* < 0.0001) by 98.97% and 86.16%, respectively.

Concerning kidney function biomarkers, intraperitoneal administration of MTX significantly increased the serum levels of creatinine by 51.94% at (*p* < 0.01) (Fig. 8c), urea and BUN by about two folds (*p* < 0.0001) (Fig. 8d and e), compared to control rats. However, pretreatment of rats with GLU decreased MTX-induced elevation in the levels of urea and BUN by 58.6% and 58.5% (*p* < 0.05), respectively. Also, co-administration of GLY with MTX reversed MTX-induced elevation in the creatinine level (*p* < 0.01), and reduced MTX-induced elevation in urea and BUN by 84.4% and 84.4% (*p* < 0.0001), respectively. Moreover, pretreatment of rats with MET decreased MTX-induced elevation in the levels of creatinine by 86.57% (*p* < 0.05), urea and BUN by 76.9% and 76.8% (*p* < 0.0001), respectively, while pretreatment of rats with LEU decreased MTX-induced elevation in urea and BUN levels by 52.8% and 52.6% (*p* < 0.01), respectively.

#### Effects of methotrexate and amino acids on hepatic and renal lipid peroxidation

**Fig. 9:**
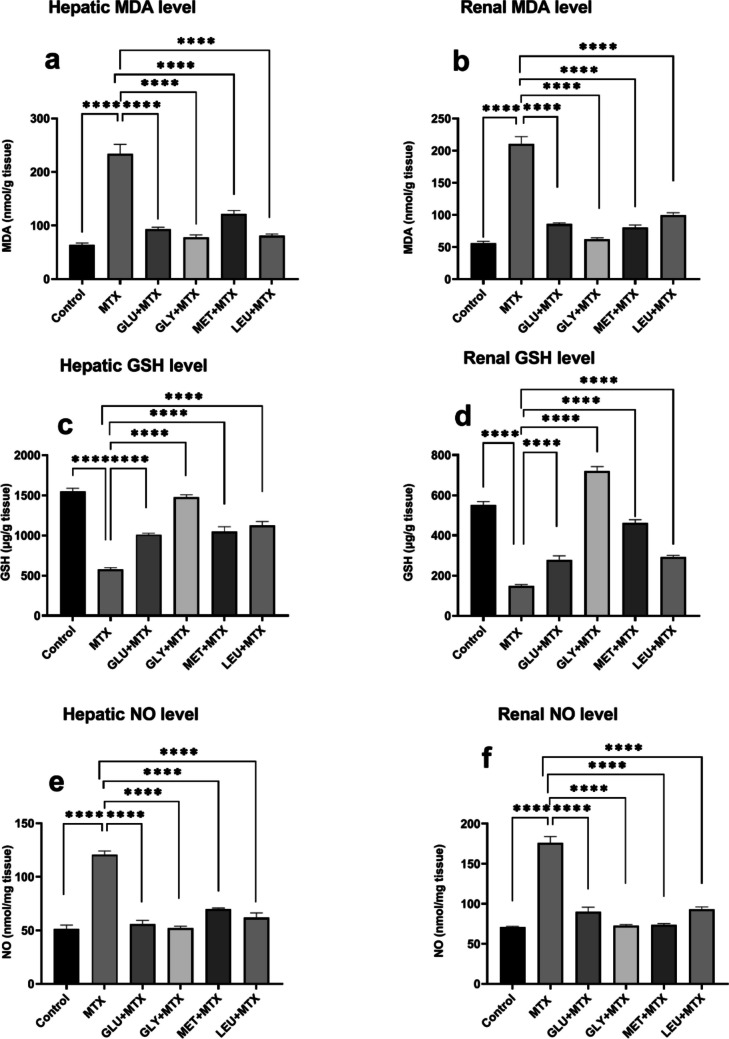
Effects of glutamine (GLU), glycine (GLY), methionine (MET), or leucine (LEU) on the MDA level in liver (**a**) and kidney (**b**), GSH in liver (**c**) and kidney (**d**) and NO levels in liver (**e**) and kidneys (**f**) of rats intoxicated with methotrexate (MTX). Values are represented as means ± SEM. *n* = 6 per group. Statistically significant difference among groups is indicated as ****p < 0.0001. Statistical analysis was done using analysis of variance (ANOVA) followed by Tukey as post hoc test for multiple comparisons

As shown in Fig. 9, MDA levels of the liver and the kidney, as products of lipid peroxidation, were significantly increased (*p* < 0.0001) by more than two folds in the liver (Fig. 9a) and the kidneys (Fig. 9b), in MTX-treated rats as compared with control ones. However, pretreatment of rats with GLU decreased the MDA level (*p* < 0.0001) by 60.24% and by 59.22% in liver and kidney, respectively, as compared with MTX-treated rats. Besides, co-administration of GLY with MTX decreased MDA levels (*p* < 0.0001) by 66.73% and 70.64% in the liver and the kidney, respectively, as compared with MTX-treated rats. In addition, pretreatment of rats with MET reduced MDA levels (*p* < 0.0001) by 47.96% and 61.83% in liver and kidney, respectively, compared to the MTX-treated group, while pretreatment of rats with LEU reduced the level of MDA (*p* < 0.0001) by 65.43% and 52.23% in liver and kidney, respectively, compared with MTX-treated rats.

#### Different amino acids ameliorated hepatic and renal reduced glutathione in MTX-treated rats

Data in Fig. 9 demonstrates that intraperitoneal injection of MTX significantly decreased the GSH level (*p* < 0.0001) by 62.68% and 73.14% in liver (Fig. 9c) and kidney (Fig. 9d), respectively, compared to control rats. However, pretreatment of rats with GLU alleviated MTX-induced reduction in GSH level (*p* < 0.0001) by 74.26% and 32.38% in liver and kidney, respectively. Fortunately, co-administration of GLY with MTX restored GSH level in the liver and the kidneys (*p* < 0.0001) and return it to a level comparable to control ones. In addition, pretreatment of rats with MET counteracted MTX-induced decrease in the GSH level (*p* < 0.0001) by 81.47% and 77.96% in liver and kidney, respectively. Moreover, pretreatment of rats with LEU elevated GSH level that was decreased by MTX by 94.62% and 35.65% in liver and kidney, respectively (*p* < 0.0001).

#### Effects of methotrexate and different amino acids on total nitric oxide in liver and kidney in vivo

Data in Fig. 9 illustrates that intraperitoneal administration of MTX significantly increased the NO level (*p* < 0.0001) in rats’s liver (Fig. 9e) and kidneys (Fig. 9f) by more than one fold, compared to control rats, while pretreatment of rats with GLU decreased MTX-induced elevation of NO (*p* < 0.0001) by 93.67% and 81.79% in liver and kidney, respectively. Fortunately, co-administration of GLY with MTX reversed the increase in NO level in both the liver and the kidneys and restored them near control values (*p* < 0.0001). In addition, pretreatment of rats with MET decreased MTX-induced elevation in NO (*p* < 0.0001) by 72.93% and 97.38% in the rat’s liver and kidneys, respectively, while pretreatment of rats with LEU decreased MTX-induced elevation in NO (*p* < 0.0001) by 84.65% and 78.26% in the liver and kidney, respectively.

#### Histopathological examination and lesion scoring

**Fig. 10:**
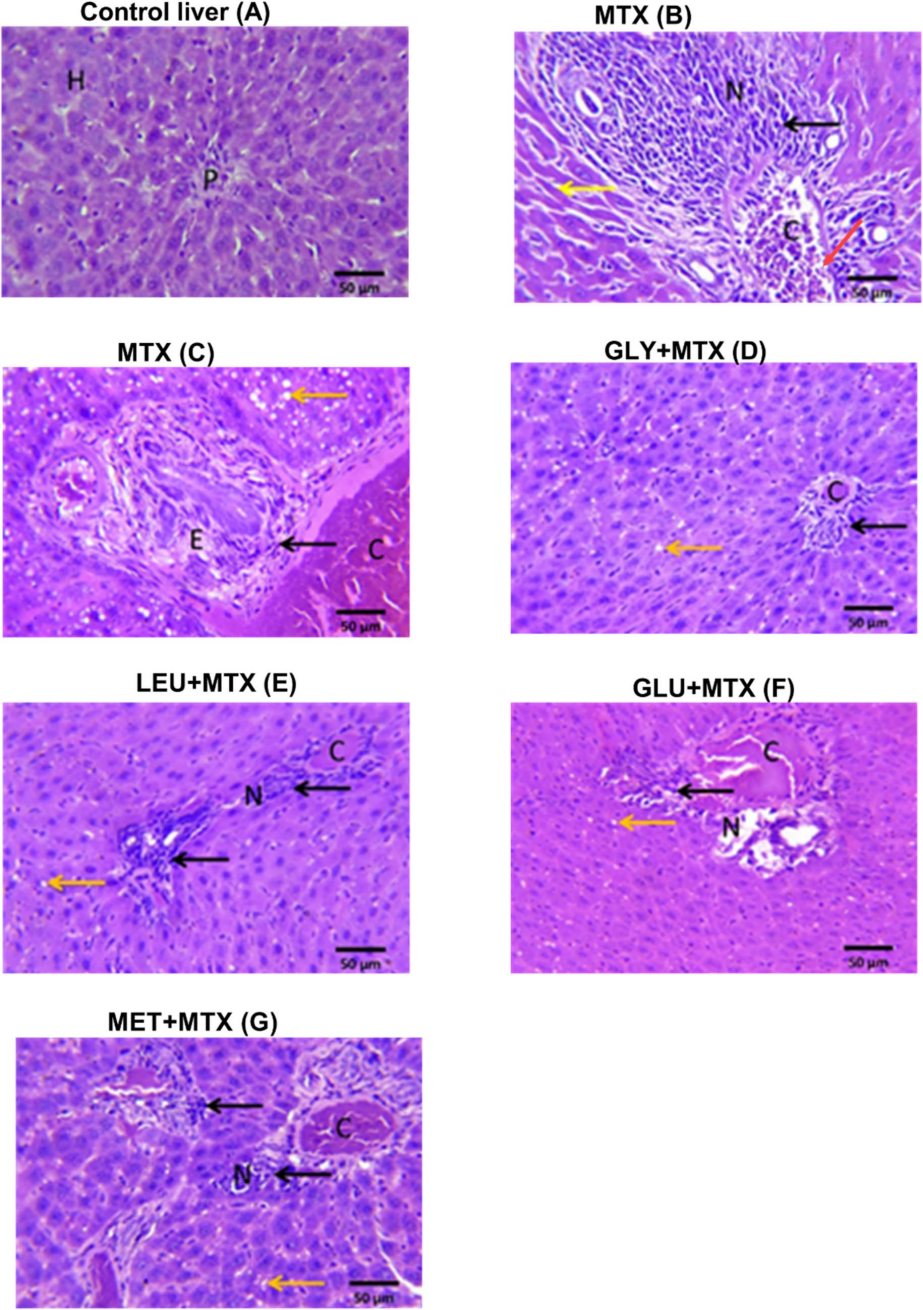
Representative photomicrographs of hematoxylin and eosin (H&E) stained sections of the livers demonstrate the effects of glycine (GLY), leucine (LEU), glutamine (GLU), or methionine (MET) on methotrexate (MTX)-induced histopathological alterations in the livers of rats. **A** shows control liver with normal histoarchitecture of the hepatocytes (H) and normal portal area (P). **B** and **C** represent MTX-treated liver MTX, show severe liver injury. Typical histopathological signs including hepatocellular necrosis (N), severe vascular congestion (**C**), atrophied hepatic cord (yellow arrow), vacuolation of the hepatocytes (orange arrow), mononuclear inflammatory cell infiltrations (black arrow), congested sinusoids (red arrow), and edema (**E**). (**D**, **E**, **F**, and **G**) represent rats treated with GLY, LEU, GLU, and MET, respectively, showing improvements in the liver histopathological alterations induced by MTX to varying levels

Results obtained from histopathological examination provided supportive evidence for the biochemical analysis. Concerning liver tissue, the control group (Fig. 10A) showed a normal histoarchitecture of the hepatocytes and normal portal area, whereas MTX-treated animals showed severe histopathological alterations including severe vacuolation of the hepatocytes, severe hepatocellular necrosis, congested sinusoids, edema, atrophied hepatic cord, severe vascular congestion, and portal inflammation with severe mononuclear inflammatory cells infiltrations (Fig. 10B and C). On the contrary, rats pre-treated with GLY showed normal liver histology except for mild vascular congestion, mild hepatocytes vacuolation, and mild mononuclear inflammatory cell infiltrations (Fig. 10D). Also, pretreatment with LEU (Fig. 10E), demonstrated obvious improvement in liver histopathology as compared to the MTX group. Livers of LEU group showed mild vascular congestion, mild vacuolation of the hepatocytes, mild hepatocellular necrosis, and moderate mononuclear inflammatory cells infiltrations. Besides, treatment with GLU resulted in a significant reduction in the MTX-induced pathological lesions (Fig. 10F), livers showed moderate vascular congestion, mild vacuolation of the hepatocytes, mild hepatocellular necrosis, and moderate mononuclear inflammatory cells. Moreover, MET (Fig. 10G) showed mild protection of liver cells from MTX-induced injury. Tissues had moderate vascular congestion, mild vacuolation of the hepatocytes, moderate hepatocellular necrosis, and moderate mononuclear inflammatory cells infiltrations. The histopathological scoring of liver tissues in different experimental groups is demonstrated in Table 1.

**Table 1:** Histopathological damage scoring of hepatic tissues in different experimental groups

Histopathological change for the liver	Control	MTX	GLY + MTX	LEU + MTX	GLU + MTX	MET + MTX
Vacuolation of the hepatocytes	**-**	** + + + **	** + **	** + + **	** + + **	** + + **
Hepatocellular necrosis	**-**	** + + + **	** + **	** + **	** + + **	** + + **
Mononuclear inflammatory cell infiltrations	**-**	** + + + **	** + **	** + + **	** + + **	** + + **
Vascular congestion	**-**	** + + + **	** + **	** + **	** + + **	** + + **
Edema	**-**	** + + **	**-**	** + **	** + **	** + + **
Atrophied hepatic cord	**-**	** + + + **	**-**	** + **	** + **	** + + **

*MTX*, methotrexate; *GLY*, glycine; *LEU*, leucine; GLU, glutamine; *MET*, methionine. The severity of pathological lesions was evaluated using the following semi-quantitative scoring system as (−) absence of the lesion = normal histology with zero involvement of the examined field, (+) mild = 5–25% of the tested field was involved, (+ +) moderate = 26–50% of the examined field was involved, and (+ + +) severe damage ≥ 50% of the examined tissue sections were involved depending on the number of affected slides and regions within the same slide.

**Fig. 11:**
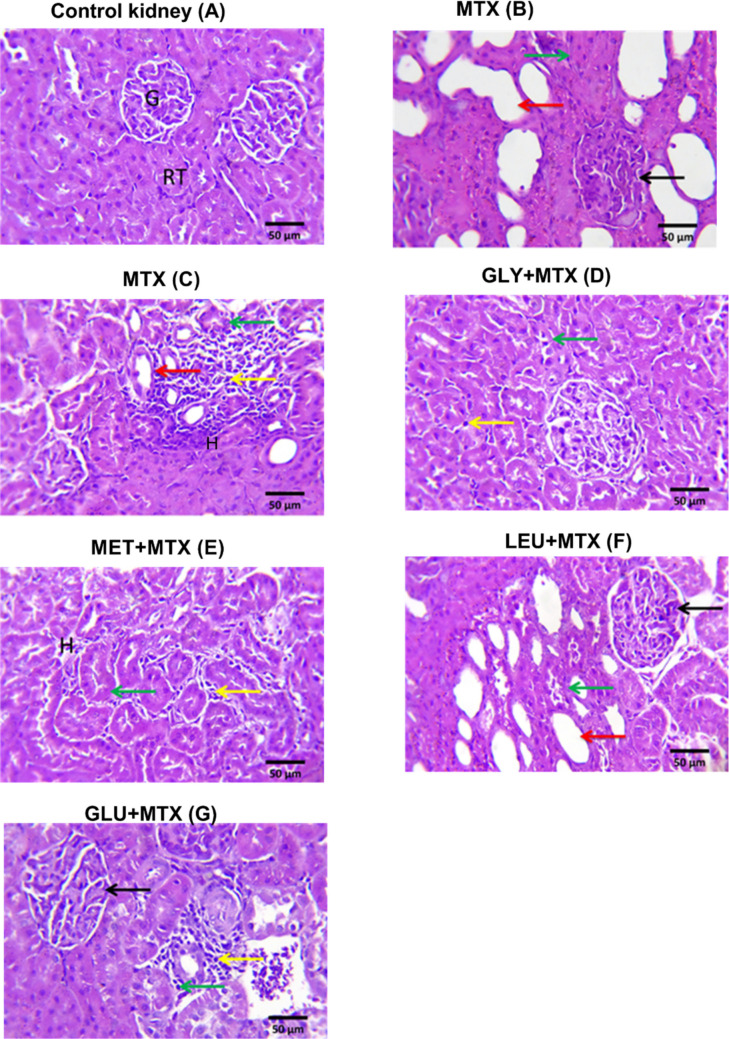
Representative photomicrographs of hematoxylin and eosin (H&E) stained sections of the kidneys demonstrate the effects of glycine (GLY), methionine (MET), leucine (LEU), or glutamine (GLU) on methotrexate (MTX)-induced histopathological alterations in the kidneys of rats. **A** shows control kidney with normal glomeruli (H) and normal renal tubule (RT). **B** and **C** represent MTX-treated kidney and show severe kidney injury. Typical pathological alterations in renal tissue including attenuated and necrotic renal tubular epithelium (green arrow), dilated renal tubular lumen (red arrow), interstitial mononuclear inflammatory cells infiltrations (yellow arrow), hypercellular glomeruli (black arrow), vascular congestion of blood vessels (**C**) and hemorrhage (**H**). (**D**, **E**, **F**, and **G**) represent rats treated with GLY, MET, LEU, and GLU, respectively, showing improvements in the kidney histopathological alterations induced by MTX to varying levels

In addition, analysis of the kidneys tissue demonstrated the control group (Fig. 11A), exhibited normal structure of the kidney with normal glomeruli and normal renal tubule, while MTX-treated rats showed severe histopathological change including severe attenuated and necrotic renal tubular epithelium, dilated renal tubular lumen, severe interstitial mononuclear inflammatory cells infiltrations, hypercellular glomeruli, severe vascular congestion, and severe hemorrhage (Fig. 11B and C). Rats pre-treated with GLY had normal kidney histology except for mild attenuated and necrotic tubular epithelium and mild interstitial mononuclear inflammatory cells infiltrations (Fig. 11D). Besides, treatment with MET showed a marked protection of kidney cells from MTX-induced injury (Fig. 11E), tissues showed mild attenuated and necrotic tubular epithelium, mild interstitial mononuclear inflammatory cells infiltrations, and mild hemorrhage. Also, rats pre-treated with LEU (Fig. 11F) had moderate improvement in kidney histopathology as compared with MTX group. Kidneys from the LEU group showed mild attenuated and necrotic tubular epithelium, dilation of the renal tubular lumens, and hypercellular glomeruli. Furthermore, treatment with GLU showed mild improvement in the MTX-induced pathological lesions in the kidney tissue (Fig. 11G); tissues showed moderate attenuated and necrotic tubular epithelium, hypercellular glomeruli, and moderate interstitial mononuclear inflammatory cells infiltrations. The histopathological scoring of renal tissues in different experimental groups is illustrated in Table 2.

**Table 2:** Histopathological damage scoring of renal tissues in different experimental groups

Histopathological change for the kidneys	Control	MTX	GLY + MTX	MET + MTX	LEU + MTX	GLU + MTX
Attenuated and necrotic renal tubular epithelium	**-**	** + + + **	** + **	** + **	** + + **	** + + **
Dilatation of the renal tubular lumen	**-**	** + + + **	** + **	** + **	** + + **	** + + **
Interstitial mononuclear inflammatory cells infiltrations	**-**	** + + + **	** + **	** + + **	** + + **	** + + **
Congestion of blood vessels	**-**	** + + **	** + **	** + **	** + **	** + + **
Hypercellular glomeruli	**-**	** + + + **	** + **	** + **	** + + **	** + + **
Hemorrhage	**-**	** + + + **	**-**	** + **	** + **	** + **

*MTX*, methotrexate; *GLY*, glycine; MET, methionine; *LEU*, leucine; *GLU*, glutamine. The severity of pathological lesions was evaluated using the following semi-quantitative scoring system as: (−) absence of the lesion = normal histology with zero involvement of the examined field, (+) mild = 5–25% of the tested field was involved, (++) moderate = 26–50% of the examined field was involved, and (+++) severe damage ≥ 50% of the examined tissue sections were involved depending on the number of affected slides and regions within the same slide

## Discussion

There is an increasing demand to understand the mechanisms underlying hepatorenal toxicity to provide measures that would minimize the occurrence of this fatal side effect by researchers (Owumi et al. 2019). MTX is an anti-metabolite drug widely used as an anti-cancer and immunosuppressive agent; however, its therapeutic efficacy is often limited due to its severe organ toxicity especially on rapidly proliferative tissues such as liver and kidneys (Howard et al. 2016; Naughton 2008). Owing to the critical roles of the liver and the kidney in the body, selecting and identifying effective agents to prevent hepatorenal toxicity of MTX are of crucial medical importance (Elsawy et al. 2021). Hereby, we demonstrated the protective effects of GLU, GLY, MET, and LEU amino acids against MTX-induced hepatorenal damage and illustrated that such protective activities are mediated through upregulation of reduced glutathione and downregulation of lipid peroxidation and nitric oxide levels as well as further inhibition of inflammatory and apoptotic pathways.

The isolated primary hepatocytes is simple, fast, reproducible, and reliable method. It is considered the gold standard for hepatotoxicity researches expressing the most typical hepatic biochemical functions, offering an initial assessment of drug toxicity and enzyme activity, as evidenced by numerous studies (Ghoneim 2009; Kaur et al. 2023; Khatab et al. 2022). Primary suspended hepatocytes preserves elevated functioning, offering a more precise link to in vivo toxicity and metabolism (Soldatow et al. 2013; Zhang et al. 2012). The current in vitro study on the isolated cultured hepatocytes showed a significant improvement in the cell viability in the groups treated with GLY, GLU, MET, or LEU before MTX as compared with cells received MTX only. Trypan blue is a simple, cheap, and reproducible cytotoxicity assay, which can be applied in vitro only enabling us testing multiple concentrations of the therapeutic agents in a short experimental time (Aslantürk 2018). Different concentrations of each amino acid were tested to find the smallest concentration cause a significant protective effect to decrease toxicity and enhance safety (Dimmitt et al. 2017).

The increase in cell viability as evidenced by trypan blue test, reflects the cytoprotective ability of these amino acids. It has been reported that GLY protects isolated rats hepatocytes against hypoxic injury (Brecht and De Groot 1994). Also, a recent study stated that GLY protected suspended hepatocytes against diclofenac-induced liver injury (Elkot et al. 2025). Turkez et al. demonstrated that GLU improved the viability of cultured rat hepatocytes treated with dioxins (Turkez et al. 2012). Moreover, LEU was able to significantly decrease endogenous protein degradation as well as, the amount of autophagic vacuoles in isolated rat hepatocytes (Grinde and Seglen 1981).

Tsuji et al. reported that MET exerted a protective effects on cultured rat hepatocytes (Tsuji et al. 1990), which is in agreement with our findings. On the contrary, another study stated that high concentration of MET induced cytotoxicity to isolated hepatocytes (Dever and Elfarra 2008). This conflicting results about MET may be attributed to the difference in the administrated concentrations. Actually, MET is considered the most toxic amino acid involved in protein synthesis process. Its toxicity is mainly attributed to MET transmethylation, in which MET is converted to S-adenosylmethionine (SAM) depending on ATP consumption. Accumulations of SAM as well as excessive ATP depletion from continuous SAM formation contribute to MET induced hepatotoxicity (Dever and Elfarra 2008; Yalçınkaya et al. 2009). Interestingly, the present results illustrated that pretreatment of suspended hepatocytes with MET (12.5 mM) prior to MTX exhibited a considerable cytoprotective effect.

Renal slices provide a valuable bridge between studies on isolated cells and whole animals as it contain all cell types present in kidney in vivo, in their normal cellular interactions (Baverel et al. 2013). Besides, it provide a physiologically relevant, multicellular, and ethically favorable in vitro system which preserve tissue integrity and allowing controlled assessment of renal functions and toxic response. Unlike isolated cell cultures, renal slices maintain the physiological gradients of oxygen, enzymes, and metabolites. This provides a closer approximation to in vivo renal metabolism and toxicity (Obatomi et al. 1998; Zhang et al. 1992). The determination of LDH leakage in the culture medium of renal slices is a reliable, simple, and fast biological marker for kidney damage. LDH release into a medium is an index of irreversible cell necrosis (a type of cell death) (Aslantürk 2018), as well as disintegration of the cell membrane and enzyme leakage (Formigli et al. 2000). In the present study, treatment of the renal slices with MTX (100 µM) significantly increased the LDH release from the cells, suggesting loss of the renal cells integrity (Zhang et al. 1992), in accordance with previously reported studies (Helal and Said 2020; Owumi et al. 2019).

The present findings demonstrated that groups pre-treated with GLY, GLU, LEU or MET exhibited a significant decrease in LDH release compared with the MTX-only group. Mahran et al. illustrated that GLY acted as a nephron-protector against cisplatin-induced nephrotoxicity and restored LDH release in kidney slices (Mahran et al. 2011). In addition, GLU attenuated cisplatin-induced renal tubular injury in vitro via different mechanisms including maintaining viability and cell membrane integrity (Kim et al. 2015). Besides, Vahdatpour et al. reported that di-peptides containing GLY and LEU prevented kidney alterations in diabetic mice (Vahdatpour et al. 2020). Moreover, a previous study that MET ameliorated polymyxin-induced nephrotoxicity in rats (Azad et al. 2018).

Several hypotheses have been proposed regarding the mechanisms underlying MTX toxicity, including apoptosis, inflammation, and oxidative stress (El-Sheikh et al. 2015). In the present study, we investigated the potential protective activities of the four amino acids considering these mechanisms. Undeniably, apoptosis plays an essential role in several hepatic and renal diseases (Elsawy et al. 2021). Caspase-3 is an aspartate specific cysteine protease, known for its executioner role in the apoptotic pathways (intrinsic and extrinsic). It is an important marker of irreversible apoptosis. Once activated, it enhances the cleavage of several important cellular proteins (Mazumder et al. 2008). In the present study, MTX treatment significantly upregulated caspase-3 levels in both suspended hepatocytes and renal slices, thus indicating an induction of apoptotic cell death in the liver and kidneys. This is consistent with previous reports on hepatic and renal tissues in vitro and in vivo (El-Sheikh et al. 2015; Elsawy et al. 2021; Khatab et al. 2022; Owumi et al. 2019). However, the marked reduction in caspase-3 activity in suspended hepatocytes and renal slices in the groups pre-treated with the GLY, GLU, LEU, or MET signifies their anti-apoptotic properties to varying degrees.

In line with the present results, previous reports pointed out that GLY attenuated the induced apoptosis in rodents through down-regulation of caspase-3 and other pro-apoptotic proteins (Elkot et al. 2025; Jacob et al. 2003; Zhang et al. 2020). Consistent with our findings, several reports stated that GLU ameliorated apoptosis in liver and kidneys of rodents by inhibiting apoptosis induced either by intracellular stimuli or by exogenous agents (Kim et al. 2015; Matés et al. 2002; Szijártó et al. 2007; Thomas et al. 2022). Agreeing with our results, di-peptides synthesized from GLY and LEU protected the liver of diabetic mice against apoptosis-related changes (Mesgari-Abbasi et al. 2022).

The present data showed that MET down-regulated caspase-3 levels in suspended hepatocytes and renal slices, too. As mentioned before, there is a great controversy about the role of MET in protecting or harming the liver cells. Several investigations have illustrated that feeding rats with MET-deficiency diets causes liver injury induced by several mechanisms including apoptosis; however, supplying the animals with constant MET diet protects the liver and inhibits apoptosis-related changes (Caballero et al. 2015; Veskovic et al. 2019). On the other hand, Yalçınkaya, S., et al. stated that rats fed with high MET diet for long period develop apoptotic changes in the liver through upregulation of pro-apoptotic proteins and downregulation of anti-apoptotic proteins (Yalçınkaya et al. 2009). These conflicting results may be attributed to different doses, method of administration and treatment period. Our results indicate that MET may ameliorate apoptosis in liver and kidney tissues at specific concentrations and doses.

MTX has been shown to enhance production of pro-inflammatory cytokines such as (TNF-α) which stimulates cellular pathology (Conway and Carey 2017). TNF-α stimulates cytokine cascade release and exacerbates neutrophil-mediated tissue injury through its effects on endothelial cells. Moreover, TNF-α activation increases nitric oxide production, which aggravates oxidative damage and contributes to cell death (Zhang and An 2007). The present results demonstrated that MTX intoxication markedly increased the levels of TNF-α in cultured hepatocytes and renal slices, in agreement with previous studies (Elsawy et al. 2021; Khatab et al. 2022; Owumi et al. 2019). The elevation in TNF-α level signifies an induction of inflammation in the liver and kidney tissues of MTX-treated groups. The inflammatory effect of MTX was further confirmed by histopathological analysis, which revealed severe mononuclear inflammatory cells infiltrations in the liver and kidney tissues. On the other hand, the marked decrease in TNF-α levels in the groups pre-treated with amino acids indicates their anti-inflammatory properties.

It was reported that glycine administration decreases TNF-α level in intoxicated rat’s liver by blocking calcium influx in isolated Kupffer cells (Roth 2007). In addition, GLY modified the pro-inflammatory profiles of patients with type II diabetes (Cruz et al. 2008). A recent study reported that GLY decreased TNF-α level in isolated rat’s hepatocytes intoxicated with diclofenac (Elkot et al. 2025). Consistent with our results, several studies showed that GLU downregulated TNF-α level and other pro-inflammatory cytokines in liver and kidney injury models (Schemitt et al. 2019; Su et al. 2021; Thomas et al. 2022). In agreement with the present finding, LEU decreased hepatic steatosis and adipose tissue inflammation in diabetic mice by down-regulating of TNF-α level (Macotela et al. 2011). In addition, LEU and other BCAAs attenuated TNF-α level in the liver of CCL_4_-intoxicated rats (Khedr and Khedr 2017). In line with the present results, MET attenuated MTX-induced nephrotoxicity in rats via different mechanisms including downregulation of TNF-α (Abdel-Wahab et al. 2023).

Following completing in vitro experiments, we performed in vivo model in order to guarantee the fulfillment of our aims. The evaluation of the enzyme activities in the body fluids and tissues is vital in the screening and diagnosis of cellular injury and different diseases (Ozer et al. 2008). Actually, serum enzymes and tissue biomarkers are considered sensitive indicators of cellular damage by xenobiotics before confirmation by histological examination (Owumi et al. 2019). Serum enzymes were measured depending on their specific cellular locations. ALT and AST are enzymes present normally in large amount in the liver. However, in pathological conditions such as membrane injury or hepatocyte necrosis, these enzymes are released into the circulation and their serum levels are elevated (Ozer et al. 2008). Therefore, the significant elevation in the rat’s serum aminotransferases levels following MTX administration is an indicator of disrupted membrane permeability associated with hepatic damage which suggested that liver injury model was successfully established. This elevations induced by MTX intoxication are agreed with previous studies (Abdel-Daim et al. 2017; Al-Azem et al. 2019; Bu et al. 2018). However, the marked reduction in the levels of serum AST and ALT in rats pretreated with amino acids suggests their protective effects against MTX-induced liver damage to varying degrees.

In line with our results, GLU decreased the serum levels of ALT and AST in previous studies (Schemitt et al. 2016, 2019). Also, GLY protected the liver of mice from injury and reduced ALT and AST levels near to the control values (Zhang et al. 2020). Mesgari-Abbasi et al. stated that dipeptides and tripeptides containing LEU significantly reduced the levels of liver enzymes in diabetic mice (Mesgari-Abbasi et al. 2022). Besides, LEU included in a mixture of BCAAs improved the biomarkers of liver function in intoxicated rats (Khedr and Khedr 2017). Moreover, MET significantly decreased serum ALT and AST levels in intoxicated-rats (Onaolapo et al. 2017).

The markers of renal functions (serum urea, creatinine, and BUN) play a key role in the assessment of kidney functions and the diagnosis of kidney injury. Urea, creatinine, and BUN act as biomarkers of the glomerular function, and their elevation indicates renal impairment (Elsawy et al. 2021). Based on the findings obtained from the present study, there was a significant increase in urea, creatinine and BUN levels in MTX-intoxicated rats as reported previously (Abdel-Daim et al. 2017; El-Sheikh et al. 2015; Owumi et al. 2019). In all amino acid-treated groups, urea and BUN levels were significantly decreased compared to MTX-intoxicated group, while the creatinine level was significantly decreased only in the groups treated with glycine or methionine compared to MTX-treated rats. In the present study, GLY effectively attenuated the renal functional damage induced by MTX to a level comparable to healthy rats, in agreement with a previous study (Shafiekhani et al. 2019). Besides, previous reports stated that, MET reduced the serum creatinine and urea levels in different kidney injury models (Abdel-Wahab et al. 2023; Onaolapo et al. 2017).

On the other hand, the present results demonstrated that pretreatment of rats with GLU before MTX couldn’t significantly reduce the level of serum creatinine. This is in line with a previous study which stated that GLU could not prevent cisplatin-induced elevation in plasma creatinine levels or decrease in creatinine clearance (de Oliveira Mora et al. 2003). May be after increasing the dose of GLU, the results would get better, because previous studies showed that GLU at higher doses attenuated kidney function tests including urea and creatinine (Kim et al. 2015; Su et al. 2021).

Moreover, pretreatment with LEU could not prevent MTX-induced elevation in the creatinine level. To the best of your knowledge, this is the first study to investigate the effect of intraperitoneal administration of LEU at 50 mg/kg for 6 days on MTX-induced renal toxicity indices. However, Pillai, S. M., et al. reported that excess (BCAAs) including LEU, in diet for weeks, interfered with renal function of rats, causing a reduction in the glomerular filtration rate and enhancing renal fibrosis via affecting the energy metabolism, while the levels of excreted electrolytes and urea were comparable across all groups receiving different diets (Pillai et al. 2019). The authors attributed their findings to the fact states that BCAAs metabolism feeds into β-oxidation and the citric acid cycle and so competes with fatty acid metabolism. In addition, BCAAs modulate PI3-AKT signaling and its downstream transcriptional regulation such as transcription factor KLF15 (Kruppel-like factor 15) (Liu et al. 2017; Pillai et al. 2019).

To elucidate the hepatorenal protective mechanism of the four amino acids in rats, we explored the role of their administration on the hepatic and renal antioxidant defense systems. Evaluating an antioxidant agent is controlled by many factors such as the bioavailability that should be taken into consideration. Therefore, the capacity and efficacy of antioxidants may be assessed most accurately in vivo by analysis of biological fluids and tissues from experimental animals (Niki 2010). There is a great evidence for the role of excessive intracellular free radicals generation in mediating MTX hepatorenal toxicity (El-Sheikh et al. 2015). GSH is a well-known versatile and ubiquitous antioxidant having the ability to protect the cell via different mechanisms. These mechanisms include scavenging and destruction of free radicals, restoration of damaged molecules and maintenance of protein thiols in their reduced state (Rana et al. 2002). As illustrated in the present results, rats intoxicated with MTX showed significant reduction in hepatic and renal GSH levels which confirms its cellular depletion. This is in agreement with previous studies (Abdel-Daim et al. 2017; Elsawy et al. 2021; Helal and Said 2020). GSH depletion may be explained by its overconsumption to destruct excessive ROS produced by MTX administration. However, the increase in GSH level in liver and kidneys of the rats pretreated with the amino acids indicates that GLY, GLU, MET, and LEU enhanced GSH availability required for cells to mitigate free radicals overproduced by MTX.

Agreeing with the present results, GLY administration to ethanol-intoxicated rats restored GSH levels in the liver and brain near the control values (Senthilkumar et al. 2004). GLY maintained the GSH level in the rat’s kidney as the control values after lead intoxication (Shafiekhani et al. 2019). A recent study demonstrated that GLY restored GSH level against diclofenac-induced toxicity in isolated rat’s hepatocytes (Elkot et al. 2025). GSH is synthesized from the glycine, cysteine and glutamate, so GLY is a substrate for the GSH synthesis and the carboxy terminal glycine moiety of GSH protects the molecule from degradation (Senthilkumar et al. 2004; Szijártó et al. 2007). Besides, GLU provides the source of glutamate to this glutathione system in many locations such as liver and kidneys that may explain its ability to increase GSH levels (Matés et al. 2002). Consistent with our findings, GLU restored hepatic GSH level in vitro and in vivo models (Schemitt et al. 2019; Szijártó et al. 2007; Turkez et al. 2012). Kim et al. pointed out that GLU maintained GSH in the rat’s kidneys to a level comparable to healthy rats through reducing the toxin uptake (Kim et al. 2015).

In addition, Veskovic et al. demonstrated that mice fed with MET-deficient diet developed fatty liver disease via different mechanism including decreased GSH levels and increased MDA and NO levels (Veskovic et al. 2019). Besides, MET protected the liver of rats from injury by prevention of GSH depletion and increasing other antioxidants to scavenge excess ROS produced (Onaolapo et al. 2017). In line with our data, LEU increased the glutathione peroxidase and decreased MDA levels in the liver of piglets (Chen et al. 2019). Also, BCAAs mixture including LEU increased glutathione peroxidase in CCL_4_-intoxicated rats (Khedr and Khedr 2017). Furthermore, di- and tri-peptides containing LEU and GLY exerted strong antioxidant activities via increasing glutathione peroxidase level as well as, reducing MDA level in diabetic mice (Vahdatpour et al. 2020).

Furtherly, malondialdehyde (MDA) is the major reactive end-product of the peroxidation of membrane polyunsaturated fatty acids which affects the membrane permeability and fluidity, and as a consequence loss of the membrane integrity (Elsawy et al. 2021). The present study illustrated that MTX administration to rats increased MDA levels in liver and kidneys, in line with other previous studies (Abdel-Daim et al. 2017; Çakır et al. 2011; Helal and Said 2020). This elevation indicates that MTX enhanced a tissue damage and a breakdown of the antioxidant defense mechanisms in rats. However, the marked decrease in MDA levels following administration of the four amino acids indicates their anti-lipid peroxidative ability. The present findings showed that GLY significantly reduced MDA levels in liver and kidney as compared to MTX-intoxicated rats, in accordance with previous studies (Senthilkumar et al. 2004; Shafiekhani et al. 2019; Zhang et al. 2020). Besides, GLU significantly reduced MDA level in liver and kidneys, in agreement with many researches (Schemitt et al. 2019; Su et al. 2021; Szijártó et al. 2007). In addition, MET was able to decrease MDA levels in liver and kidneys, consistent with previous reports (Caballero et al. 2015; Veskovic et al. 2019). Moreover, LEU reduced MDA level in the liver and kidney, in line with previous studies (Chen et al. 2019; Khedr and Khedr 2017; Vahdatpour et al. 2020).

Nitric oxide (NO) plays a vital role in inflammation and is implicated in the pathophysiology of various diseases including hepatotoxicity and nephrotoxicity (Owumi et al. 2019). NO is an inhibitor of catalase activity that triggers the activity of peroxidase. Therefore, excessive production of NO causes accumulation of hydrogen peroxide that reacts with the superoxide radical and produces the hydroxyl radicals initiating lipid peroxidation (Elsawy et al. 2021). NO levels elevation increases protein nitration enhancing alterations in signal transduction pathways and subsequent protein dysfunction (Schopfer et al. 2003). MTX can up-regulate the inducible NO synthase (iNOS), therefore elevate NO levels as shown in the present results. Several studies documented similar action of MTX on NO content in rodents (Abdel-Daim et al. 2017; El-Sheikh et al. 2015; Helal and Said 2020; Owumi et al. 2019).

On the contrary, rats pretreated with amino acids showed reduced levels of NO in the liver and kidneys compared to those that received MTX only. Similarly, GLY prevented excessive production of hepatic NO, thereby preventing liver injury in rats with obstructive jaundice (Fang et al. 2003). Besides, GLU reduced hepatic NO level of rats with acute liver failure (Schemitt et al. 2019). Previous study showed that, MET reduced NO levels in brain, liver, and kidneys in intoxicated rats (Onaolapo et al. 2017). Furthermore, a previous research (Tekwe et al. 2023) reported that rats treated with LEU exhibited lower NO synthesis by endothelial cells compared to the non-treated ones.

Results obtained from histopathological examination provided supportive evidence for the biochemical findings. MTX induced severe alterations of hepatic architecture, in agreement with many reports (Abdel-Daim et al. 2017; Al-Azem et al. 2019; Çakır et al. 2011; Helal and Said 2020; Owumi et al. 2019). On the other hand, rats pretreated with amino acids showed less histological alterations in the liver tissues compared to those received MTX only. Interestingly, our histological analysis showed a marked protective effect of GLY against MTX-induced injury, in accordance with previous report (Senthilkumar et al. 2004). Consistent with a previous study (Chen et al. 2018), the current results illustrated that LEU moderately ameliorated the liver histopathological alterations induced by MTX. In line with a previous report (Schemitt et al. 2019), our findings demonstrated that GLU provided mild to moderate protection against MTX-induced histopathological changes. In addition, methionine enhanced mild improvement against histopathological changes induced by MTX-intoxication. Veskovic et al. reported that mice fed with a MET-deficient diet developed liver steatosis and inflammation (Veskovic et al. 2019).

Kidney sections from MTX-treated rats revealed severe histopathological alterations, in accordance with previous studies (Abdel-Daim et al. 2017; Al-Azem et al. 2019; Çakır et al. 2011; El-Sheikh et al. 2015; Elsawy et al. 2021; Helal and Said 2020; Owumi et al. 2019). On the contrary, rats pre-treated with amino acids exhibited markedly reduced renal alterations compared to the MTX-intoxicated group. Notably, rats that received GLY largely retained the normal renal architecture, in line with a previous report (Shafiekhani et al. 2019). In addition, in the present study, MET markedly provided protection against renal histopathological damage induced by MTX, in agreement with a previous study (Azad et al. 2018). Moreover, LEU provided a moderate protection against MTX-induced structural changes. In line with these findings, Chen et al. illustrated that LEU supplementation improved glomerulosclerosis and diabetic renal hypertrophic changes in mice (Chen et al. 2018). GLU conferred mild to moderate protection against MTX-induced kidney histopathological changes. In accordance with current results, Kim et al. reported that GLU administration to rats partially mitigated necrotic tubules and tubular casts caused by cisplatin intoxication (Kim et al. 2015).

**Fig. 12:**
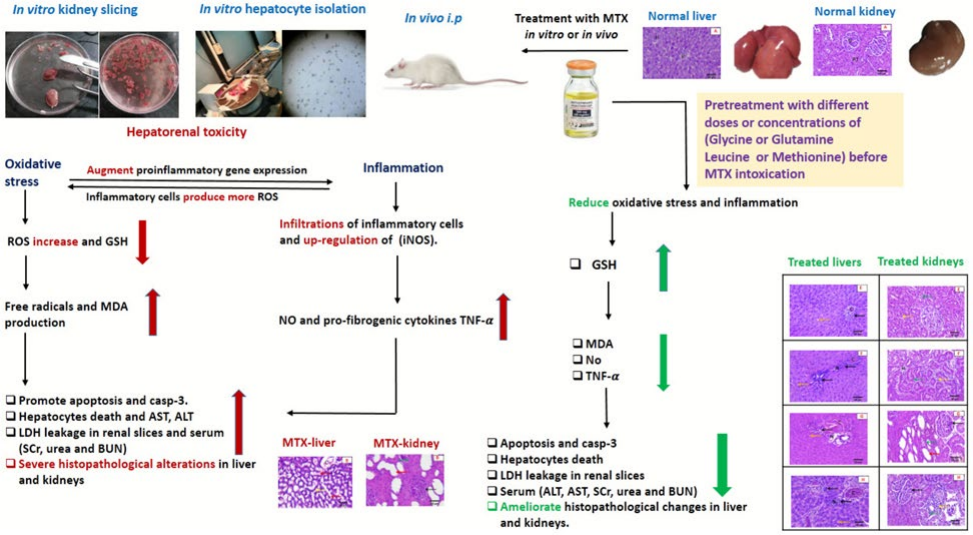
Mechanistic figure of the discussion

Despite providing valuable insights in the hepatoprotective and renoprotective effects of GLU, GLY, MET and LEU against MTX-induced hepatorenal toxicity, this study has some limitations that should be taken into consideration. Limited oxygen and nutrient diffusion to the inner layers of renal slices may cause reduced cell viability and metabolic activity during prolonged incubation. Similarly, variability in hepatocyte yield and viability may occur during isolation using the collagenase perfusion technique due to differences in enzyme activity, perfusion efficiency or tissue handling. Concerning in vivo model, the study focused on short-term biochemical and histopathological outcomes, and long-term effects of the amino acids were not evaluated. In addition, all in vivo experiments were assessed in adult male rats only; therefore, potential age or sex-related difference were not investigated (Fig. 12).

## Conclusion

In conclusion, the amino acids (GLY, GLU, LEU, and MET) exhibited protective effects against MTX-induced hepatorenal toxicity to varying degrees. The amino acids showed anti-oxidative, anti-inflammatory, and anti-apoptotic properties. The amino acids protected both liver and kidneys of rats against MTX-induced histopathological changes. Therefore, GLU, GLY, LEU, and MET may be used as adjuvant therapy to prevent MTX-induced hepatorenal toxicity, especially since these amino acids demonstrated anti-proliferative and anti-angiogenesis effects against different cancer cell lines in previous studies (two birds with one stone). Future researches should be directed towards evaluating the clinical applicability of GLU, GLY, LEU, and MET as adjuvant therapies to MTX.

## Supplementary Information

Below is the link to the electronic supplementary material.

ESM 1 (DOCX 1.20 MB)

## Data Availability

All data generated or analyzed during this study are included in this published article and its supplementary information files.

## Abbreviations

ALT: Alanine transaminase
AST: Aspartate transaminase
ANOVA: Analysis of variance
BCA: Bicinchoninic acid
BCAAs: Branched chain amino acids
BSA: Bovine serum albumin
BUN: Blood urea nitrogen
CCL_4_: Carbon tetrachloride
DMEM: Dulbecco’s modified Eagle medium
DTNB: 5,5-Dithiobis (2-nitrobenzoic acid)
FBS: Fetal bovine serum
GSH: Reduced glutathione
GLU: Glutamine
GLY: Glycine
HEPES: [N-(2 Hydroxyethyl) piperazine-N’-2 ethane sulfonic acid]
iNOS: Inducible nitric oxide synthase
KH: Krebs-Henseleit
LDH: Lactate dehydrogenase
LEU: Leucine
LP: Lipid peroxidation
MET: Methionine
MDA: Malondialdehyde
MTX: Methotrexate
NED: Naphthyl ethylenediamine dihydrochloride
NO: Nitric oxide
7-OH MTX: 7-Hydroxy methotrexate
Opm: Oscillations per minute
pNA: P-Nitroaniline
RNS: Reactive nitrogen species
ROS: Reactive oxygen species
SAM: S-adenosyl l-methionine
SNA: SUlfanilamide
TBA: Thiobarbituric acid
TMP: Tetramethoxypropane
TNF-α: Tumor necrosis factor alpha
VCL_3_: Vanadium chloride III
