# Supplementary material for: Amino acids protect against methotrexate-induced hepatorenal toxicity in vitro and in vivo
Source: Naunyn Schmiedebergs Arch Pharmacol. 2026 Feb 16;399(8):11365–88. doi: 10.1007/s00210-026-05010-8 (PMC13269434; doi:10.1007/s00210-026-05010-8)
Supplement: Supplementary file 1 — (DOCX 1.20 MB) [file 210_2026_5010_MOESM1_ESM.docx]

***Table (S1): Pilot study for determining the optimum concentration of each amino acid in hepatocyte isolation experiment"***

**Amino acids concentration for isolated hepatocytes**

| **Amino acid concentration added 30 min before MTX** | **% viability by TB test Mean ± S.E** | **P value Vs inducing MTX** | **Selected** |
| --- | --- | --- | --- |
| Control | 37.37 ± 1.520 |  |  |
| MTX | 15.85 ± 0.7307 | <0.0001 |  |
| GLU 6.25mM | 17.43 ± 0.5812 | 0.9382  ns | No |
| GLU 12.5mM | 21.15 ± 1.023 | <0.05 | No |
| **GLU 25mM** | **27 ± 1.181** | **<0.0001** | **Yes** |
| GLU 50mM | 30.6 ± 0.7024 | <0.0001 | No |
| GLY 6.25mM | 18.37 ± 1.035 | 0.5007  ns | No |
| GLY 12.5mM | 22.55 ± 1.564 | <0.05 | No |
| **GLY 25mM** | **34.5 ± 0.6745** | **<0.0001** | **Yes** |
| GLY 50mM | 35.03 ± 1.087 | <0.0001 | No |
| Met 3mM | 17.7 ± 0.5292 | 0.8714  ns | No |
| Met 6.25mM | 20.27 ± 1.185 | 0.1327  ns | No |
| **Met 12.5mM** | **24.53 ± 0.7227** | **<0.001** | **Yes** |
| Met 25mM | 22.10 ± 0.7810 | <0.05 | No |
| LEU 6.25mM | 17.53 ± 0.3756 | 0.9366  ns | No |
| LEU 12.5mM | 21.71 ± 1.519 | <0.05 | No |
| LEU 25mM | 28.34 ± 1.265 | <0.0001 | **Yes** |
| LEU 50mM | 32.72 ± 1.142 | <0.0001 | No |


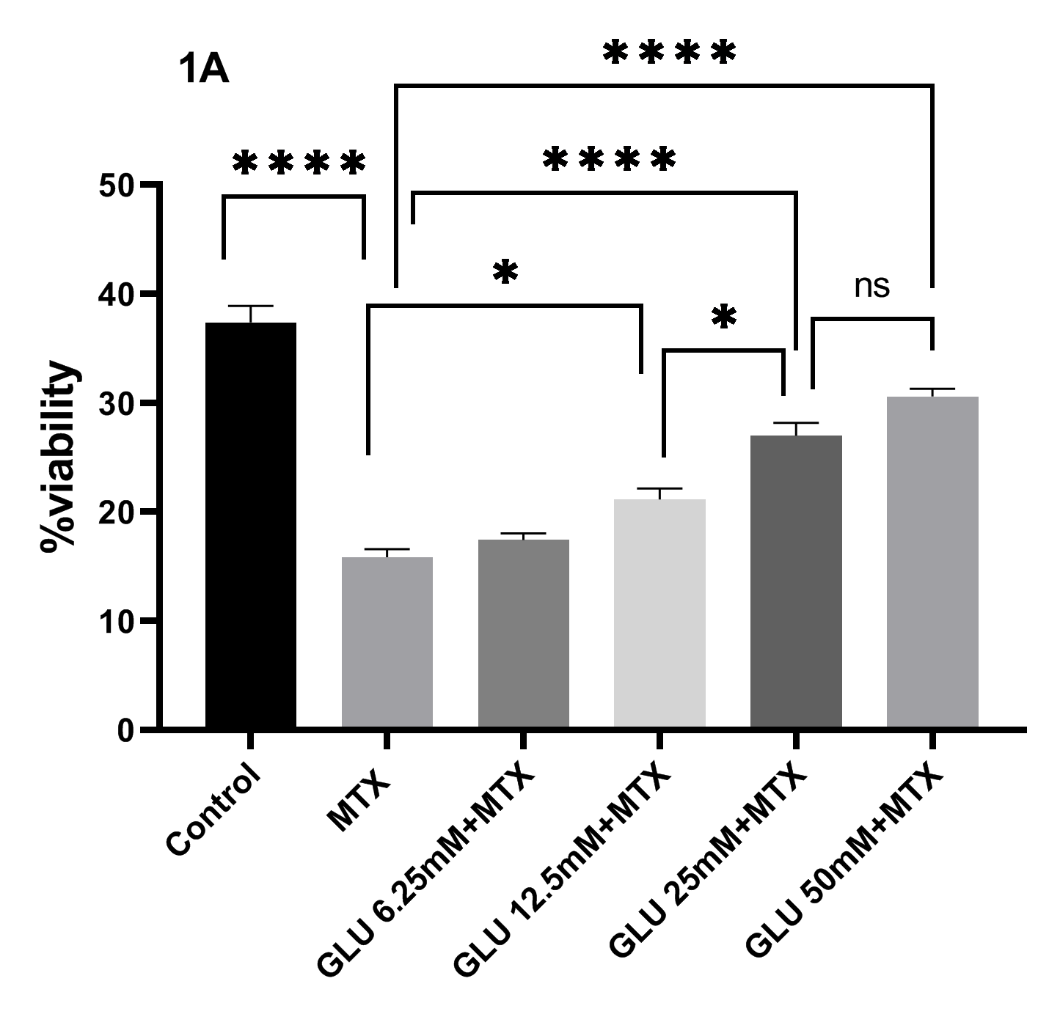


**Fig. 1** Effects of glutamine (GLU), (6.25mM, 12.5mM, 25mM and 50mM) on methotrexate (MTX)–induced trypan blue (TB) uptake in suspended hepatocytes. GLU was added 30 min before intoxication of hepatocytes with MTX. Values are represented as means ± SEM for (3–7) hepatocytes preparations. Samples were taken for TB estimation after 150 min incubation (37 °C, 100 rpm). Statistically significant difference among groups is indicated as ****p <0.0001, *p <0.05. ns: non-significant.. Statistical analysis was done using analysis of variance (ANOVA) followed by Tukey as post hoc test for multiple comparisons


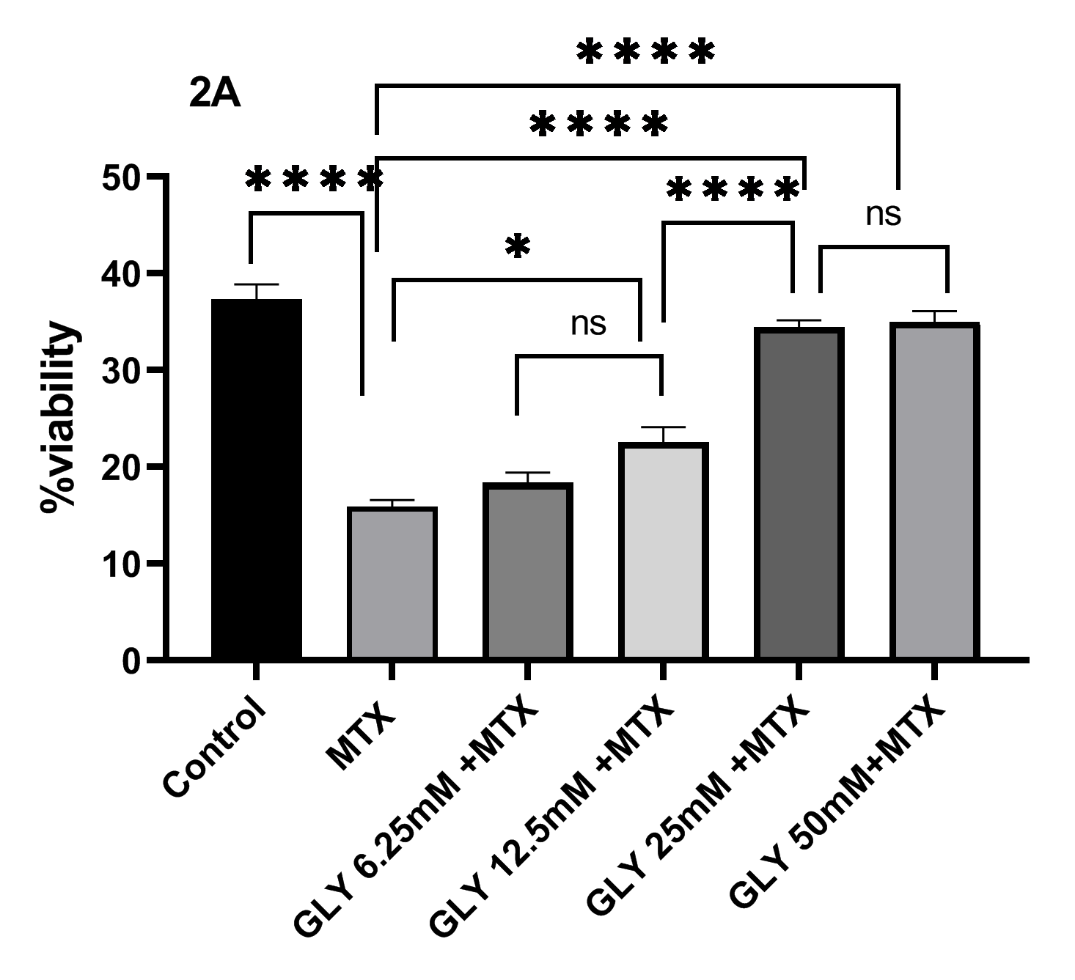


**Fig.2** Effects of glycine (GLY), (6.25mM, 12.5mM, 25mM and 50mM) on methotrexate (MTX)–induced trypan blue (TB) uptake in suspended hepatocytes. GLY was added 30 min before intoxication of hepatocytes with MTX. Values are represented as means ± SEM for (3–7) hepatocytes preparations. Samples were taken for TB estimation after 150 min incubation (37 °C, 100 rpm). Statistically significant difference among groups is indicated as ****p <0.0001, *p <0.05. ns: non-significant.. Statistical analysis was done using analysis of variance (ANOVA) followed by Tukey as post hoc test for multiple comparisons


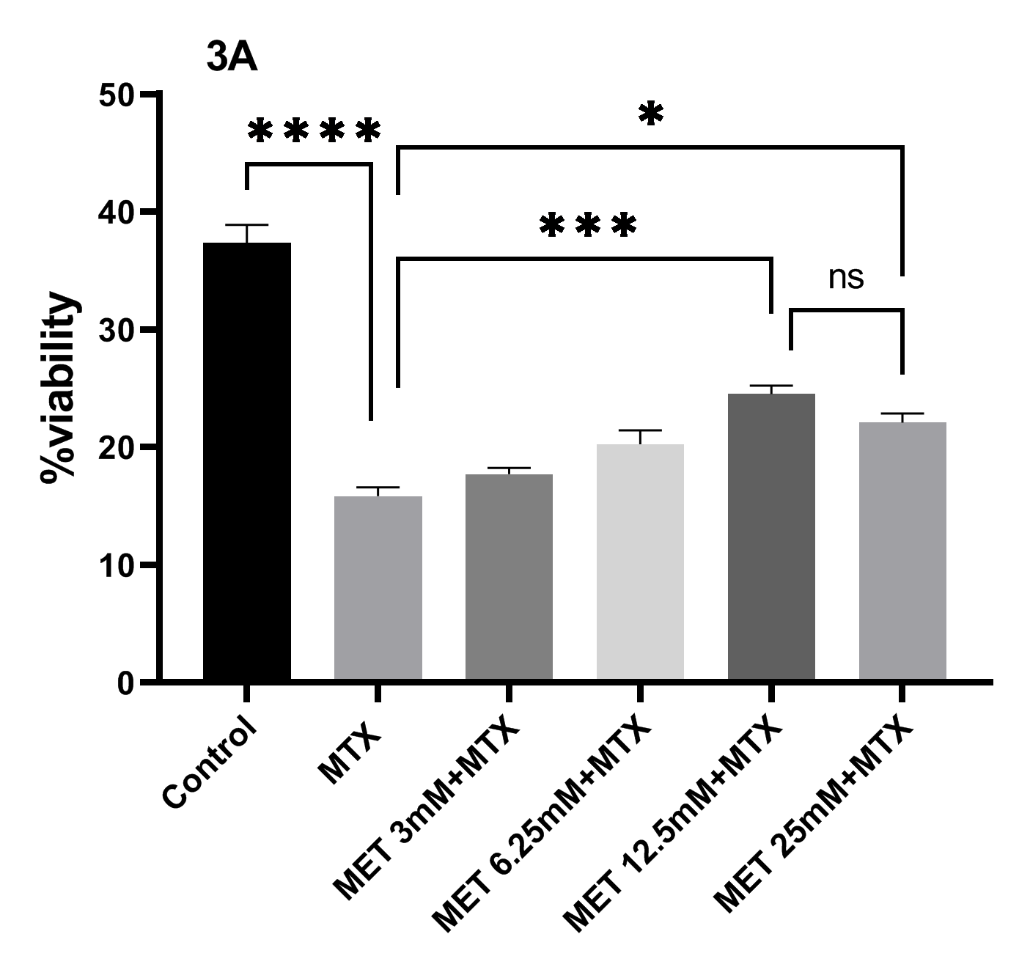


**Fig.3** Effects of methionine (MET), (3mM, 6.25mM, 12.5mM and 25mM) on methotrexate (MTX)–induced trypan blue (TB) uptake in suspended hepatocytes. MET was added 30 min before intoxication of hepatocytes with MTX. Values are represented as means ± SEM for (3–7) hepatocytes preparations. Samples were taken for TB estimation after 150 min incubation (37 °C, 100 rpm). Statistically significant difference among groups is indicated as ****p <0.0001, ***p <0.001 *p <0.05. ns: non-significant.. Statistical analysis was done using analysis of variance (ANOVA) followed by Tukey as post hoc test for multiple comparisons


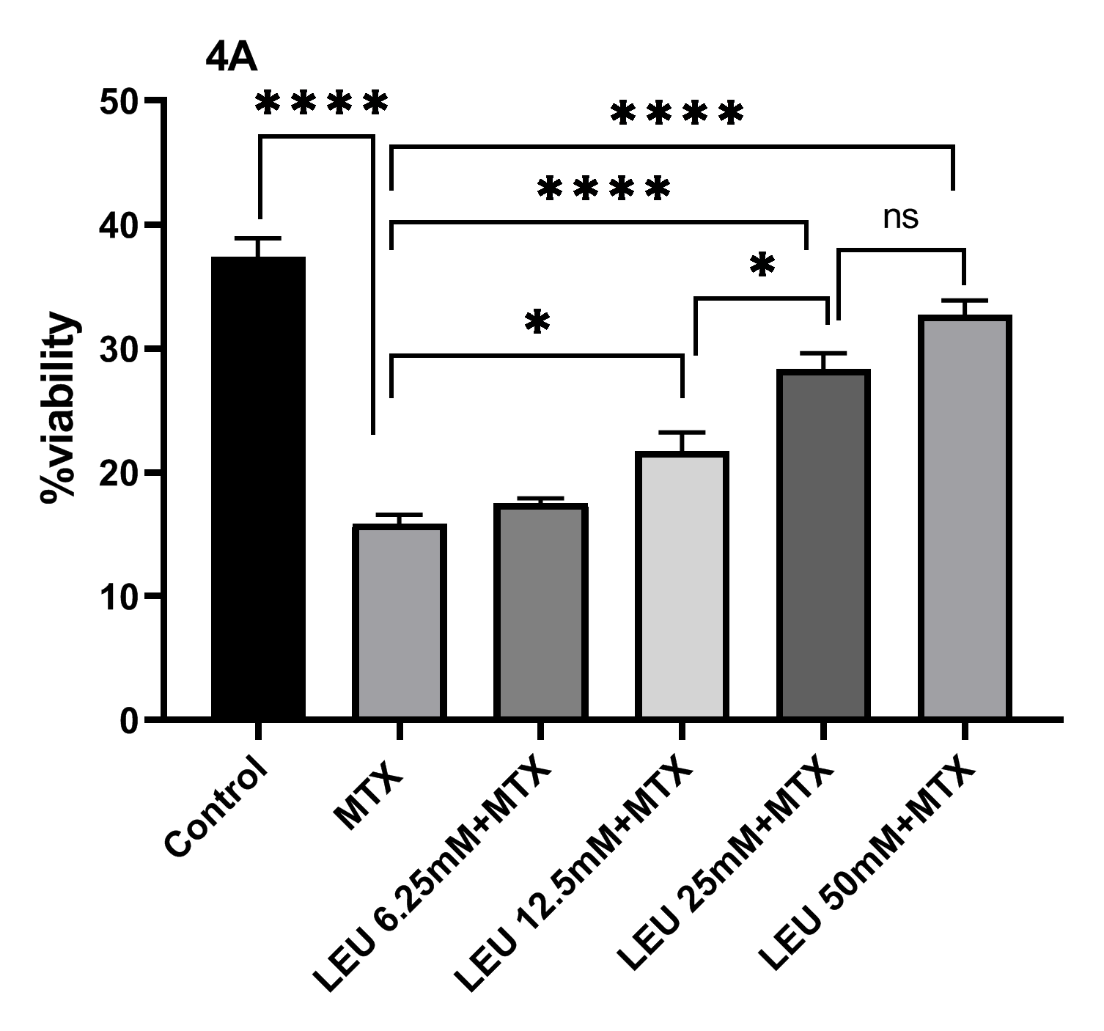


**Fig.2** Effects of leucine (LEU), (6.25mM, 12.5mM, 25mM and 50mM) on methotrexate (MTX)–induced trypan blue (TB) uptake in suspended hepatocytes. LEU was added 30 min before intoxication of hepatocytes with MTX. Values are represented as means ± SEM for (3–7) hepatocytes preparations. Samples were taken for TB estimation after 150 min incubation (37 °C, 100 rpm). Statistically significant difference among groups is indicated as ****p <0.0001, *p <0.05. ns: non-significant.. Statistical analysis was done using analysis of variance (ANOVA) followed by Tukey as post hoc test for multiple comparisons

***Table (S2): Pilot study for determining the optimum concentration of each amino acid in renal slices experiment"***

**Amino acids concentration for isolated hepatocytes**

| **Amino acid concentration added 30 min before MTX** | **LDH leakage Mean ± S.E** | **P value Vs inducing MTX** | **Selected** |
| --- | --- | --- | --- |
| Control | 136.3 ± 4.680 |  |  |
| MTX | 577.6 ± 8.016 | <0.0001 |  |
| GLU 2.5mM | 536.7 ± 12.02 | 0.1125  ns | No |
| GLU 5mM | 436.7 ± 23.33 | <0.0001 | No |
| **GLU 10mM** | **220.5 ± 4.317** | **<0.0001** | **Yes** |
| GLU 20mM | 197.3 ± 6.84 | <0.0001 | No |
| GLY 2.5mM | 424.3 ± 20.57 | <0.0001 | No |
| **GLY 5mM** | **210.5 ± 1.881** | **<0.0001** | **Yes** |
| GLY 10mM | 185.3 ± 5.419 | <0.0001 | No |
| GLY 20mM | 167.9 ± 3.526 | <0.0001 | No |
| Met 2.5mM | 364.1 ± 18.2 | <0.0001 | No |
| **Met 5mM** | **247.3 ± 5.89** | **<0.0001** | **Yes** |
| Met 10mM | 233 ± 7.44 | <0.0001 | No |
| Met 20mM | 330.6 ± 13.00 | <0.0001 | No |
| LEU 2.5mM | 552.9 ± 18.6 | 0.5887  ns | No |
| LEU 5mM | 487.1 ± 18.27 | <0.001 | No |
| **LEU 10mM** | **321.9 ± 3.901** | **<0.0001** | **Yes** |
| LEU 20mM | 289.9 ± 9.77 | <0.0001 | No |


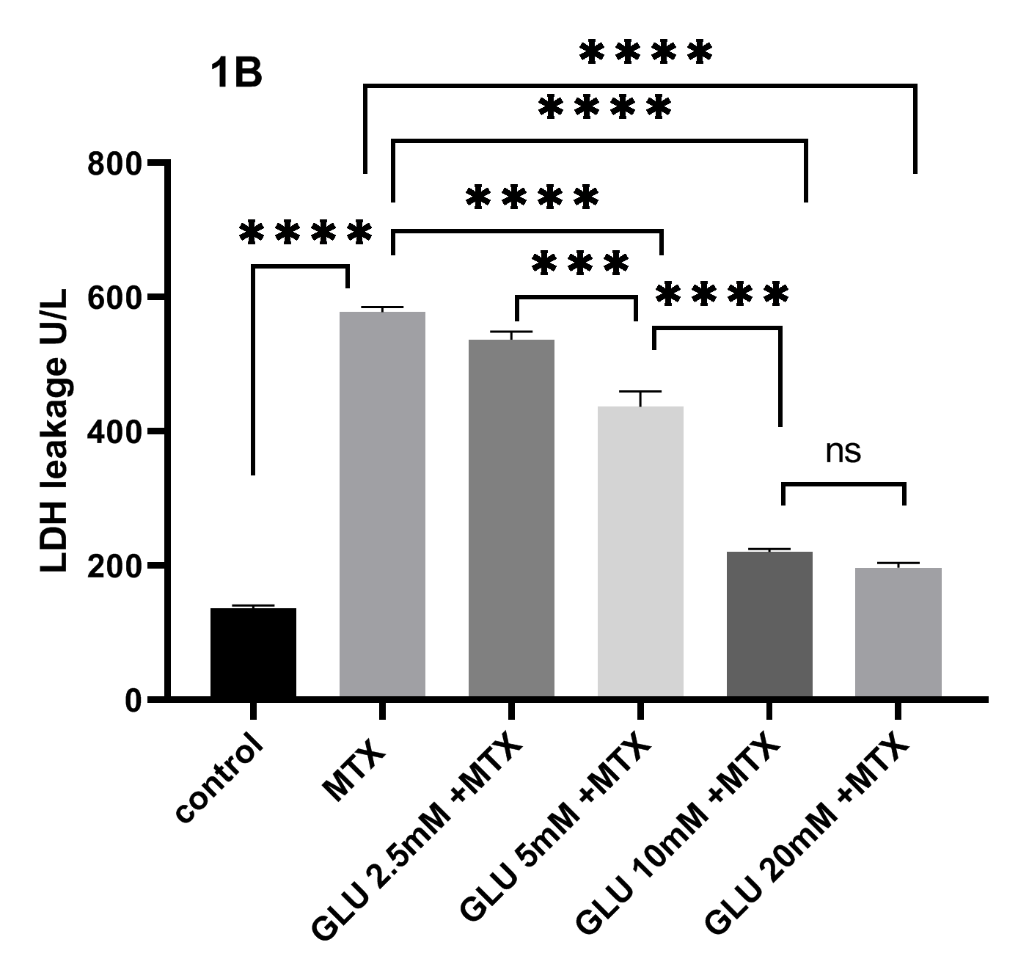


**Fig. 5** Effects of glutamine (GLU), (2.5mM, 5mM, 10mM and 20mM) on methotrexate (MTX)–induced lactate dehydrogenase (LDH) leakage in renal slices of rats. GLU was added 30 min before intoxication of renal slices with MTX. Values are represented as means ± SEM for (3–7) renal slices preparations. Samples were taken for LDH release determination after 150 min incubation (37 °C, 100 rpm). Statistically significant difference among groups is indicated as ****p <0.0001, ***p <0.001, ns: NON-significant. Statistical analysis was done using analysis of variance (ANOVA) followed by Tukey as post hoc test for multiple comparisons


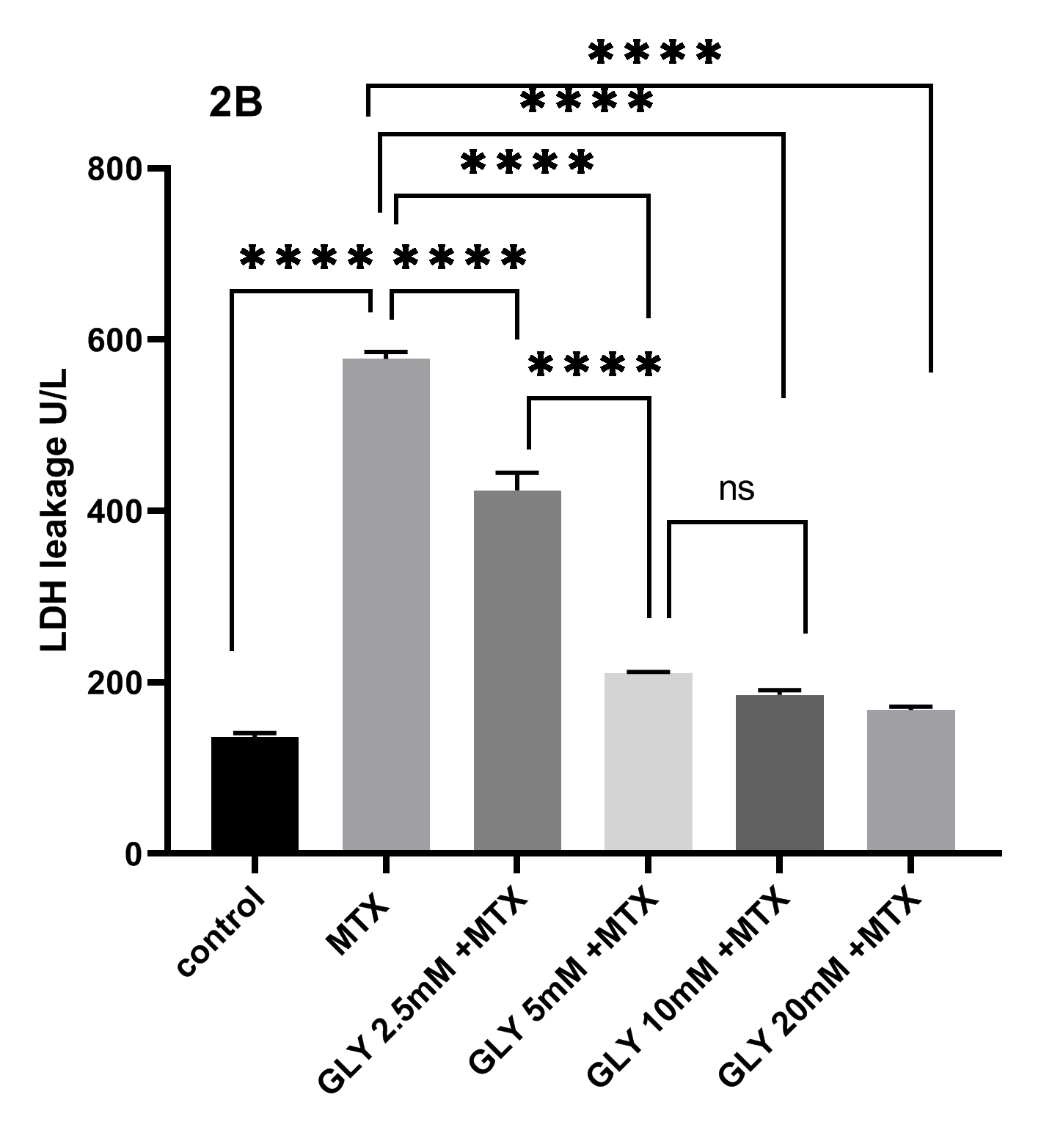


**Fig. 6** Effects of glutamine (GLY), (2.5mM, 5mM, 10mM and 20mM) on methotrexate (MTX)–induced lactate dehydrogenase (LDH) leakage in renal slices of rats. GLY was added 30 min before intoxication of renal slices with MTX. Values are represented as means ± SEM for (3–7) renal slices preparations. Samples were taken for LDH release determination after 150 min incubation (37 °C, 100 rpm). Statistically significant difference among groups is indicated as ****p <0.0001, ns: non-significant. Statistical analysis was done using analysis of variance (ANOVA) followed by Tukey as post hoc test for multiple comparisons


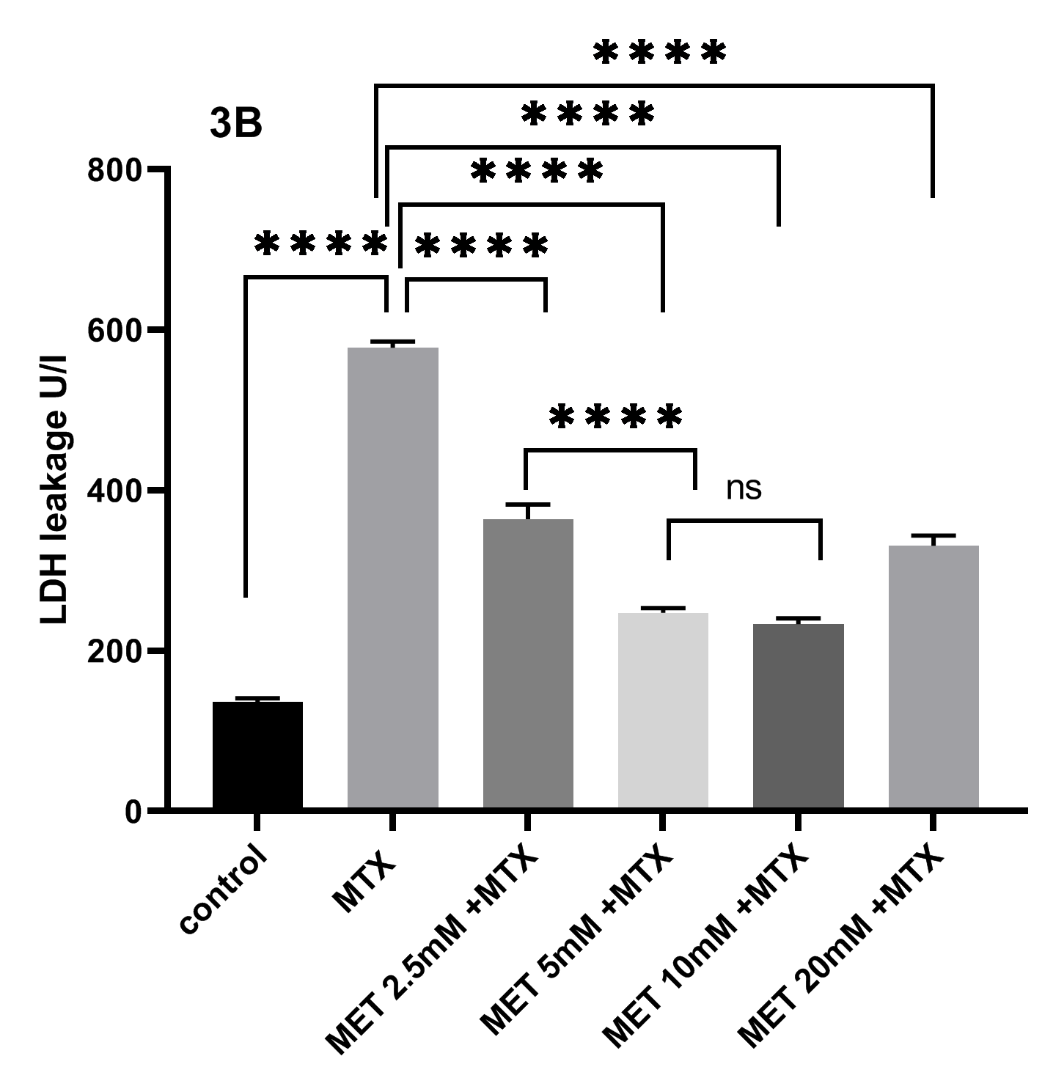


**Fig. 7** Effects of methionine (MET), (2.5mM, 5mM, 10mM and 20mM) on methotrexate (MTX)–induced lactate dehydrogenase (LDH) leakage in renal slices of rats. MET was added 30 min before intoxication of renal slices with MTX. Values are represented as means ± SEM for (3–7) renal slices preparations. Samples were taken for LDH release determination after 150 min incubation (37 °C, 100 rpm). Statistically significant difference among groups is indicated as ****p <0.0001, ns: non-significant. Statistical analysis was done using analysis of variance (ANOVA) followed by Tukey as post hoc test for multiple comparisons


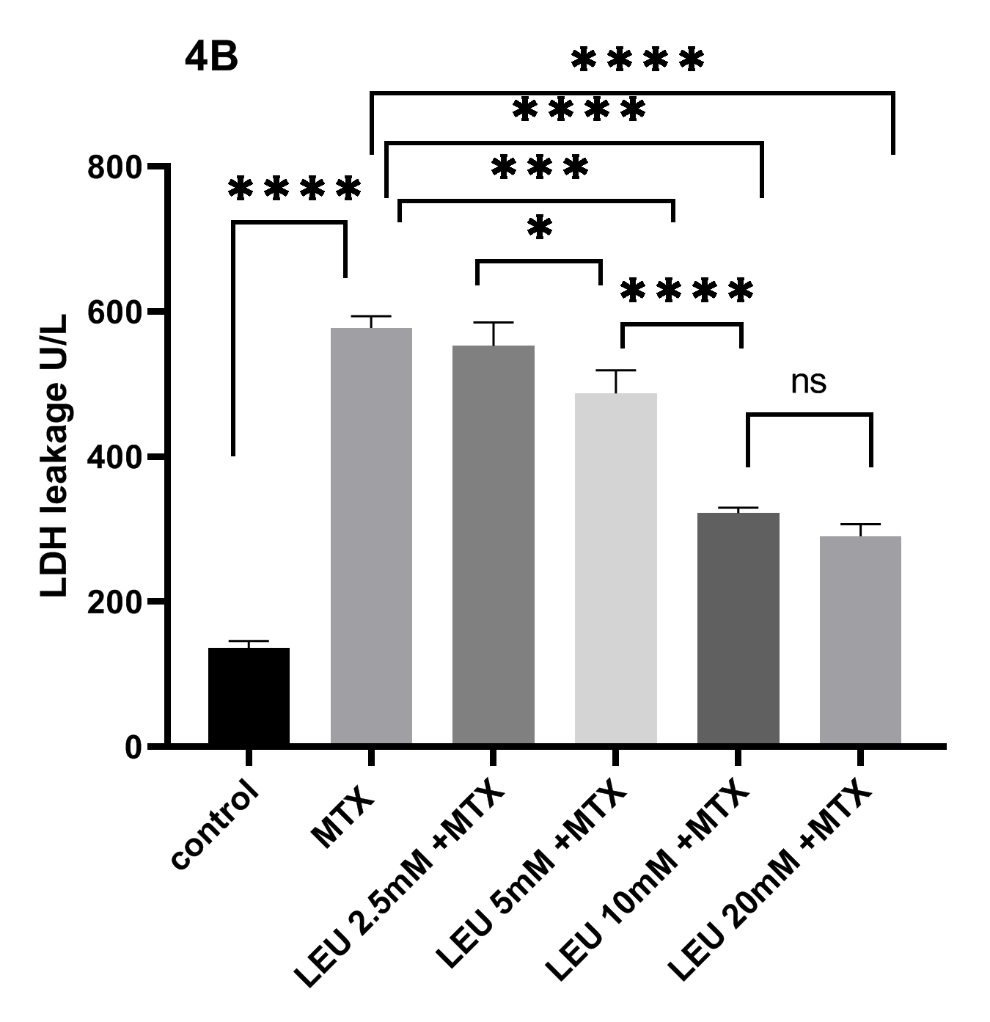


**Fig. 8** Effects of leucine (LEU), (2.5mM, 5mM, 10mM and 20mM) on methotrexate (MTX)–induced lactate dehydrogenase (LDH) leakage in renal slices of rats. LEU was added 30 min before intoxication of renal slices with MTX. Values are represented as means ± SEM for (3–7) renal slices preparations. Samples were taken for LDH release determination after 150 min incubation (37 °C, 100 rpm). Statistically significant difference among groups is indicated as ****p <0.0001, ***p <0.001, *p <0.05 ns: non-significant. Statistical analysis was done using analysis of variance (ANOVA) followed by Tukey as post hoc test for multiple comparisons.
